# At-home wireless monitoring of acute hemodynamic disturbances to detect sleep apnea and sleep stages via a soft sternal patch

**DOI:** 10.1126/sciadv.abl4146

**Published:** 2021-12-22

**Authors:** Nathan Zavanelli, Hojoong Kim, Jongsu Kim, Robert Herbert, Musa Mahmood, Yun-Soung Kim, Shinjae Kwon, Nicholas B. Bolus, F. Brennan Torstrick, Christopher S. D. Lee, Woon-Hong Yeo

**Affiliations:** 1 George W. Woodruff School of Mechanical Engineering, College of Engineering, Georgia Institute of Technology, Atlanta, GA 30332, USA.; 2 Center for Human-Centric Interfaces and Engineering at the Institute for Electronics and Nanotechnology, Georgia Institute of Technology, Atlanta, GA 30332, USA.; 3 Huxley Medical Inc., Atlanta, GA 30318, USA.; 4 Wallace H. Coulter Department of Biomedical Engineering, Parker H. Petit Institute for Bioengineering and Biosciences, Georgia Institute of Technology and Emory University, Atlanta, GA 30332, USA.; 5 Institute for Robotics and Intelligent Machines, Neural Engineering Center, Flexible and Wearable Electronics Advanced Research, Institute for Materials, Georgia Institute of Technology, Atlanta, GA 30332, USA.

## Abstract

Obstructive sleep apnea (OSA) affects more than 900 million adults globally and can create serious health complications when untreated; however, 80% of cases remain undiagnosed. Critically, current diagnostic techniques are fundamentally limited by low throughputs and high failure rates. Here, we report a wireless, fully integrated, soft patch with skin-like mechanics optimized through analytical and computational studies to capture seismocardiograms, electrocardiograms, and photoplethysmograms from the sternum, allowing clinicians to investigate the cardiovascular response to OSA during home sleep tests. In preliminary trials with symptomatic and control subjects, the soft device demonstrated excellent ability to detect blood-oxygen saturation, respiratory effort, respiration rate, heart rate, cardiac pre-ejection period and ejection timing, aortic opening mechanics, heart rate variability, and sleep staging. Last, machine learning is used to autodetect apneas and hypopneas with 100% sensitivity and 95% precision in preliminary at-home trials with symptomatic patients, compared to data scored by professionally certified sleep clinicians.

## Teaser

A systematic study of soft materials and flexible mechanics yields a soft patch capable of detecting sleep stages and sleep apnea.

## INTRODUCTION

One of the more poignant tragedies in modern medicine is that many pathologies with highly effective treatments remain undiagnosed (*1*). For instance, obstructive sleep apnea (OSA) is well controlled through continuous positive airway pressure, but only 20% of the 30 million Americans with OSA are diagnosed, and this problem is exacerbated in marginalized communities (*2*). OSA occurs when breathing is interrupted because of airway obstruction, and the accumulation of carbon dioxide in the bloodstream triggers chemoreceptors to produce sympathetic arousals that trigger a fight or flight response throughout the body (*3*). The resultant degradation of sleep quality and accumulation of stress in the cardiovascular system can greatly affect patient health, especially in the presence of common comorbidities (*2*, *4*). The American Academy of Sleep Medicine (AASM) has cited work by Frost and Sullivan indicating that undiagnosed OSA carries an economic burden of $150 billion dollars, and this does not include decreases in quality of life and life expectancy (*2*, *5*). Despite the dire need for improved OSA diagnostics, current devices and recently reported alternatives in the literature are limited by several key challenges (*1*, *5*, *6*). First, traditional in-laboratory polysomnography (PSG) is too expensive and low throughput to diagnose 30 million subjects (*1*). Second, current U.S. Food and Drug Administration (FDA)–approved home OSA tests involve complicated systems with multiple parts and are composed of rigid materials that interface poorly with the skin (*1*, *6*). As a result, clinical throughput is limited by patient training, user or device error results in around 10% of tests failing, substantial time segments are rejected for analysis because of poor signals, multinight tests are difficult because of obtrusive measurements, and nonrepresentative sleep is recorded (*1*). Because of these limitations, numerous alternative methods have been proposed, including electrocardiography (ECG), thoracic acoustics, mandible movement, chest vibrations, photoplethysmography (PPG), and bioimpedance (*7*–*20*).

None of these systems, however, can provide clinicians with all the clinically relevant metrics outlined by the AASM in a single, user-friendly patch (*1*, *5*). Specifically, the AASM requires that sleep apnea detection devices assess sleep time, cardiovascular function, oxygen saturation (SpO_2_), body position, respiratory effort (RE), and respiration-induced physiological changes in a schema termed SCOPER (sleep, cardiovascular, oximetry, position, effort, and respiratory) (*5*). Table 1 provides a summary of recently reported wearable devices to detect apneas and the degree to which these devices meet the SCOPER criteria. Overall, OSA cannot be diagnosed or treated solely by reporting the number of apneas, but instead, a complete characterization of the subject’s physiological response to OSA is required (*21*). Even in the most sophisticated PSG systems, the impact of OSA on cardiac mechanics is inferred only through surrogate signals, like the ECG and PPG, not measured directly. For the many patients with OSA who also suffer from cardiac comorbidities, like congestive heart failure, arrythmia, and left ventricular hypertrophy, this omission can greatly complicate proper diagnosis and treatment (*5*). Therefore, meeting the SCOPER criteria is a minimum prerequisite for apnea diagnosis, but additional insights into the cardiovascular function are of great interest for the future generation of diagnostic tools.

**Table 1.** Performance comparison of home sleep monitoring devices. ACC, gross acceleration; PPG, photoplethysmogram; ECG, electrocardiogram; RF, respiratory flow; SCG, seismocardiogram; MM, mandibular movement; BI, bioimpedance; BCG, ballistocardiogram; MA, mechano-acoustics; S, sleep; C, cardiovascular; O, oximetry; P, position; E, effort; R, respiratory; RCNN, residual convolutional neural network; KNN, k-nearest neighbors; LSTM, long short-term memory; FFNN, feedforward neural network; SVM, support vector machine; CNN, convolutional neural network.

**Reference**	**Detectable** signals*	**Device** **location**	**Form factor/** **telemetry**	**Sleep SCOPER criteria^†^**						**Automated A/H** **classification** **algorithm^‡^**	**Total subjects** **(healthy/** **symptomatic)**	**Sensitivity/** **precision (%)**
				**S**	**C**	**O**	**P**	**E**	**R**			
This work	ECG, PPG, SCG, and ACC	Sternum	Soft/wireless	✔	✔	✔	✔	✔	✔	RCNN	9	100/95
											(4/5)	
(*7*)	MM	Chin	Rigid/wireless	X	X	X	✔	✔	X	Proprietary sunrise algorithm	376	88/92
											(46/330)	
(*8*)	ECG and ACC	Pectoral	Flexible/ wireless	X	✔	X	✔	✔	✔	RE threshold	110	90/94.1
											(0/110)	
(*9*)	ACC, PPG, and RF	Finger, wrist, and nasal canal	Rigid/wired	X	✔	✔	✔	X	✔	KNN	8	95
											(3/5)	
(*10*)	BI, ECG, and ACC	Sternum	Rigid/wireless	X	✔	X	✔	✔	✔	LSTM	25	68/66
											(8/17)	
(*11*)	ECG	Chest	Rigid/wired	X	X	X	X	✔	✔	LSTM	86	96/96
											(0/86)	
(*12*)	BCG and ACC	Sternum	Rigid/wireless	X	X	X	X	✔	✔	FFNN	5	97/97
											(0/5)	
(*13*)	ECG	Chest	Rigid/wired	✔	✔	X	X	✔	✔	Cardiopulmonary threshold	205	86/85
											(49/156)	
(*14*)	ECG	Chest	Rigid/wired	X	X	X	X	✔	✔	Cardiopulmonary threshold	68	100/81
											(26/42)	
(*15*)	ECG	Sternum	Flexible/wired	X	✔	X	X	X	✔	SVM	241	88/61
											(44/197)	
(*16*)	ECG	Chest	Rigid/wired	X	✔	X	X	X	✔	SVM	35	73/73
											(0/35)	
(*17*)	MA and ACC	Suprasternal notch	Soft/wireless	✔	✔	X	✔	✔	✔	–	8	–
											(8/0)	
(*18*)	MA and ACC	Trachea	Rigid/wired	✔	X	X	✔	✔	X	CNN	1852	76/90
											(262/1590)	
(*19*)	ECG	Chest	Rigid/wired	X	X	X	X	✔	✔	CNN	60	83/90
											(0/60)	
(*20*)	BI, ECG, and ACC	Sternum	Rigid/wired	X	✔	X	✔	✔	✔	Decision tree	3	74/74
											(0/3)	

*List of detectable signals: ACC, PPG, ECG, RF, SCG, MM, BI, BCG, and MA.

†Sleep SCOPER criteria: sleep, cardiovascular, oximetry, position, effort, and respiratory.

‡List of automated apnea/hypopnea classification algorithms: RCNN, KNN, LSTM, FFNN, SVM, and CNN.

In this work, we demonstrate that a wireless, all-in-one, wearable soft sternal patch can directly measure the acute hemodynamic consequences of OSA with patients at home, enabling accurate capture of all the SCOPER requirements in a single, highly user-friendly patch. This is achieved with highly conformal soft materials optimized to capture the highly sensitive sternal PPG signal and fine seismic chest vibrations 50 times lesser than gravitational acceleration, nanomembrane ECG electrodes capable of long duration, highly biocompatible electrophysiologic monitoring, advanced signal processing techniques, and time-series machine learning to elucidate physiological changes that manifest during obstructive apnea and wake, rapid eye movement (REM), and non-REM (NREM) sleep. We study theoretical and computational models to predict the mechanical device properties that lead to improved signal quality. Likewise, very-small-amplitude seismocardiography (SCG) vibrations require excellent conformal contact between the device and skin for optimal signal transduction. This is achieved through the use of highly flexible soft electronics and validated both in vitro and in vivo. Next, thin, dry gold ECG electrodes are patterned in fractal patterns to alleviate motion artifacts induced by strain and designed for long-duration use without biocompatibility challenges. We use differential pulsatile reflectance between red (660 nm) and infrared (880 nm) light to elucidate the percentage of oxyhemoglobin in the blood. ECG R-peak detection is used to determine heart rate (HR) and HR variability (HRV). Low-frequency accelerations are processed to capture tidal chest movement during respiration and body position. SCG signals segmented by the R-peak from the ECG are ensemble-averaged and processed to determine the heart’s beat by beat mechanics. These signals are analyzed via a feedforward neural network (FFNN) to classify sleep into the wake, NREM, and REM stages. The ability to analyze the heart’s mechanical performance allows for excellent differentiation of each class compared to previous works. Last, a residual convolutional neural network (RCNN) is implemented to detect apneas and hypopneas based on the sympathetic innervation of the heart upon apnea termination and the presence of disturbed respiration. The studies into device mechanics, signal transduction and processing, and machine learning provide a previously unexplored paradigm in assessing respiratory disturbances by targeting the specific mechanical, electrical, and optical properties that change during OSA. Overall, a set of these capabilities allows a single, highly user-friendly patch to produce all the essential SCOPER metrics. The fundamental studies into PPG recording from the sternum are valuable in recording this essential signal from an anatomical region traditionally deemed unfavorable to target. These studies are not valuable solely in detecting OSA but instead are of general interest for hemodynamic assessments for cardiac monitoring.

## RESULTS

### Design, functions, and skin-contact mechanics of a soft sternal patch

**Fig. 1.**
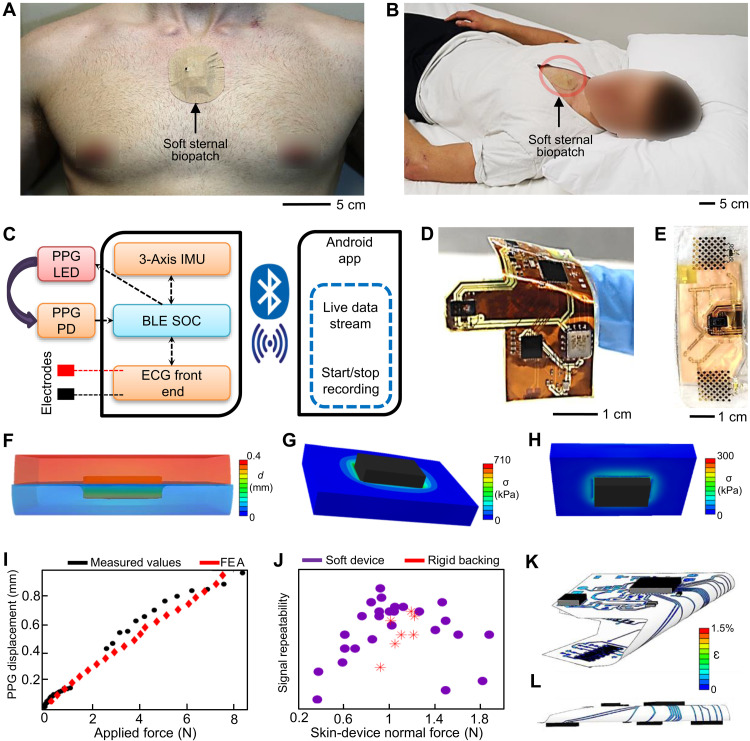
Overview of design, functions, and skin-contact mechanics of a soft sternal patch. (**A**) Photo of a soft biopatch mounted on the sternum. (**B**) Photo of a subject who is wearing the soft biopatch during sleep. (**C**) Diagram that captures the key sensing components of the biopatch for a wireless data recording with a portable device. IMU, inertial motion unit; PD, photo-detector. (**D**) Image of a soft biopatch (front-side view). (**E**) Image of the biopatch in (D) with the back-side view that faces the skin, showing a pair of nanomembrane electrodes and PPG units. (**F** to **H**) Finite element analysis (FEA) results showing the chip-embedded device, pressed to the skin (F), applied stress on the skin (G), and applied stress on the substrate (H). (**I**) Comparison of experimental and FEA results that show changes of PPG displacements to the skin as a function of an applied force. (**J**) Signal repeatability as a function of skin-device normal force for a soft device and a rigid device. (**K** and **L**) FEA results of the biopatch during bending [top view (K) and side view (L)]. LED, light-emitting diode.

This study develops a soft, wireless device capable of recording ECG, SCG, and PPG from a single location on the sternum (Fig. 1), enabling accurate capture of all the SCOPER requirements in a single, highly user-friendly patch. Sternal ECG is a well-studied and mature mechanism by which one can gain insights into the electrical performance of the heart (*11*, *19*, *22*). Here, we integrate nanomembrane, stretchable gold patterns into dry electrodes in the soft device to improve the signal-to-noise ratio (SNR) with motion and create excellent conformal contact over multiple hours (*22*, *23*). SCG is a rapidly growing field aiming to capture the cardiac vibrations that propagate through the body and result in three-dimensional seismic disturbances on the chest surface (*24*). By ensuring conformal contact between the soft device and the skin, we minimize transduction errors in this ultrasensitive vibration (*25*). The combined assessment of ECG and SCG allows for a thorough assessment of the electrical and mechanical function of the cardiovascular system during OSA (*24*). Disturbed breathing creates excess CO_2_ concentrations in the blood, and chemoreceptors trigger acute sympathetic arousal that manifests in tachycardia and sinus arrhythmia in the ECG signal (*3*). Likewise, sympathetic arousal affects the cardiac mechanics through peripheral vasoconstriction, increased venous return, and increased myocardial contractility (*3*). Pleural pressure is also markedly reduced during apnea, leading to increased venous return and transmural diastolic aortic pressure (*26*). In both cases, increased venous return increases right ventricular end diastolic volume, which decreases left ventricular compliance (*27*). This leads to decreased left ventricular end diastolic volume and ultimately a decrease in left ventricular stroke volume (*3*, *27*). In combination, the sympathetic and mechanical stresses on the heart during an apnea event will decrease pre-ejection period (PEP), left ventricular ejection timing (LVET), and the magnitude of aortic opening kinetic energy (|AO|), although these effects are less pronounced in the presence of hypertension (*3*, *27*). These physiological mechanisms are further described in fig. S1. Last, PPG is a thoroughly studied and accepted method for obtaining estimations of SpO_2_, but it is challenging to capture from the sternum because of poor basal perfusion in the cutaneous microcirculation (*28*). SpO_2_ estimation is highly dependent on PPG SNR, so optimizing device mechanics for intimate contact despite rhythmic chest movement proved essential (*29*).

The soft sternal patch device consists of an ultrathin flexible circuit on an elastomeric membrane with integrated components and nanomembrane electrodes (details of the device fabrication in fig. S2). The device adheres to the sternum both through the natural van der Waals forces at the elastomer-skin interface and pressure applied by a prestrained uniaxially stretchable tape (Kinesio Tape) as shown in Fig. 1 (A and B). The maximal delamination force was measured to be 6.7 N (fig. S3). Furthermore, the resulting circuit is highly conductive, solderable, and patternable with high spatial resolution, allowing traditional electronics to be integrated into a soft, highly flexible device. This allows for the sophisticated functionality illustrated in Fig. 1C. Figure 1 (D and E) shows the device’s top view, bending on a finger, and bottom view, nanomembrane electrodes. The low nonconformal motion of the PPG unit relative to the skin is required to achieve high-quality SpO_2_, and optimized bending stiffness alone is insufficient for quality optical signal transduction. Here, we offset the PPG module from the bottom of the board by 0.4 mm and use a tape over the device to apply downward pressure and maintain consistent pressure during motion while sleeping. This method of force application was preferred over a compressive belt or shirt because these methods are prone to complications when the subject moves at night or rolls side to side. Finite element analysis (FEA) was used to determine the optimal applied pressure, material properties, and PPG offset. The FEA results, summarized in Fig. 1 (F to H), show displacements of the device, applied stress on the skin, and applied stress on the substrate based on the uniform force (1.1 N). In this model, the skin and device exhibit high conformal contact, part of the PPG unit is embedded in the skin, and the device deforms around the remainder of the PPG unit. Motion artifacts occur when a skin acceleration fails to produce an equal acceleration in the board, causing any sensors on the board to lose coupling with the skin. Because the sensor is well embedded in the skin at an offset height of 0.4 mm after applying the downward force from the tape, this offset height was determined for the final design.

During sleep, the skin acceleration is expected to be driven primarily by bending, wave propagation, lateral translation, and torsion. The board easily matches concave bending because the skin pushes directly on the board, especially in high-body mass index (BMI) individuals. Still, convex bending requires the transmission of tensile stress through the bottom adhesive. In this study, however, the equilibrium between applied pressure and the normal skin force is disrupted as the skin pulls away at the device edges, creating a downward force independent of the bottom adhesive. Similar phenomena are observed for wave buckling. When the skin sheers, the PPG unit compresses the skin area in contact with the plane normal to the direction of travel, and this force greatly increases mechanical conformality. Likewise, disproportionate rotation is limited by the low device bending stiffness, the applied pressure at the device edges, and the skin compression and elongation required as the PPG unit rotates through the skin. The computational models were validated through video imaging of the device mechanics, and the qualitative hypotheses were validated through in vivo assessments of signal repeatability. Figure 1I compares PPG displacements into a skin-like biomimetic mold after applying a uniformly distributed load on the board surface. The details of the experimental data with a rigid circuit and a soft device appear in figs. S4 and S5 and movies S1 (soft device) and S2 (rigid device).

**Fig. 2.**
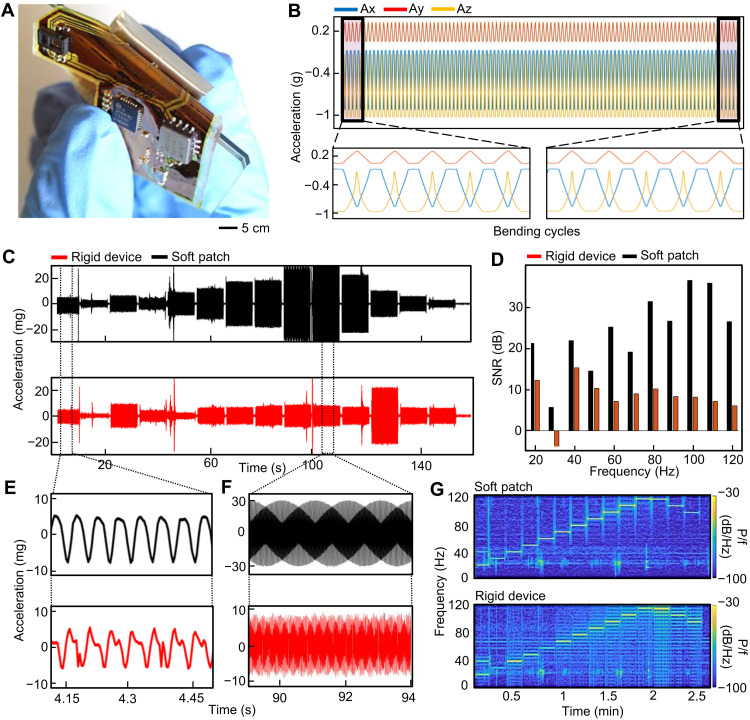
Mechanical assessment of a soft sternal patch. (**A**) Image of a soft patch with an applied bending with a radius of 3 mm. (**B**) Three-axis acceleration data plotted during 100 rounds of cyclic bending, demonstrating that the signal is identical at the beginning (bottom left) and end (bottom right) of the trial. (**C**) Comparison of acceleration data between a soft patch and a rigid board, when excited by a speaker applying tones with stepwise frequency through a biomimetic skin model. (**D**) SNR comparison of two devices in the experiment. (**E** and **F**) Zoomed-in view of modulated sign waves captured by the soft device compared to the rigid system: (E) pure tone and (F) modulated tone. (**G**) Spectral contents of the recorded signals for both devices, demonstrating a clear increase in signal fidelity with the soft patch.

The empirical results for PPG displacement into the skin are delineated into three regimes. First, the PPG unit is linearly pressed into the skin at very low pressure. Second, the device compresses to fully enclose the PPG unit and contact the skin from 0.5 to 1.5 N. Last, the PPG displacement increases linearly with high applied force after the device is fully compressed. In this study, the optimal pressure was determined to be near the end of the compressive regime because the stabilizing forces on the PPG are maximized without collapsing the cutaneous microcirculation or requiring prohibitively aggressive adhesive forces. This hypothesis was tested in human subjects by determining the signal predictability as a function of applied pressure (results in Fig. 1J and fig. S6). Signal repeatability is defined by overlaying each PPG beat after dynamic time warping, determining the beat average, and calculating the total root mean squared distance of the beats from this average. This process is illustrated in fig. S7. In the experiment, the signal repeatability increases linearly with applied pressure until optimal compression is achieved. Once the pressure is further increased, the signal repeatability becomes highly variable, likely because of restricted blood flow or artifacts transmitted through the tape under higher strain. The same soft device with a thin, rigid plastic piece affixed underneath exhibited highly diminished signal quality. It could come from lever action, poor conformal contact, or uneven force distribution. Ensemble averages of these PPG signals under different loading conditions are provided in fig. S8. Another key advantage of the soft circuit is that it demonstrates excellent bendability for conformal lamination on the skin because the two Cu layers and three polyimide (PI) layers are less than 20 μm in total thickness. This is shown in the FEA analysis of the device’s bendability (Fig. 1, K and L) and is validated empirically by cyclic bending to 180° with a 3-mm radius over 100 cycles. An image of the device during bending is provided in Fig. 2A, and the experimental results are provided in Fig. 2B. The acceleration data quality is not degraded over time, indicating that the device remains undamaged during cyclic bending. Last, the device conformality was demonstrated in an experiment where the system was placed on a biomimetic skin with a simulated single or complex tone acoustic excitation. The device was adhered to the biomimetic skin with a double-sided adhesive, and a rigid breakout board with the same accelerometer and tape was used as a comparison (fig. S9). The acceleration data for various excitations are provided in Fig. 2C, and the resultant SNR values are compared in Fig. 2D. The soft device is capable of high-quality signal transduction (SNR > 30) for small (10 to 20 mg) acoustic vibrations, whereas the rigid device is not sufficiently conformal to the skin. This is further demonstrated in Fig. 2 (E and F), which shows recorded signal morphologies for each system. Figure 2G shows the power spectral density of each device’s signals. The rigid device exhibits highly increased noise, which is visible in the nonperiodic waveforms and nonlocalized power spectral content.

### Study of physiological data measured by an all-in-one, soft sternal patch with multifunctional sensors

**Fig. 3.**
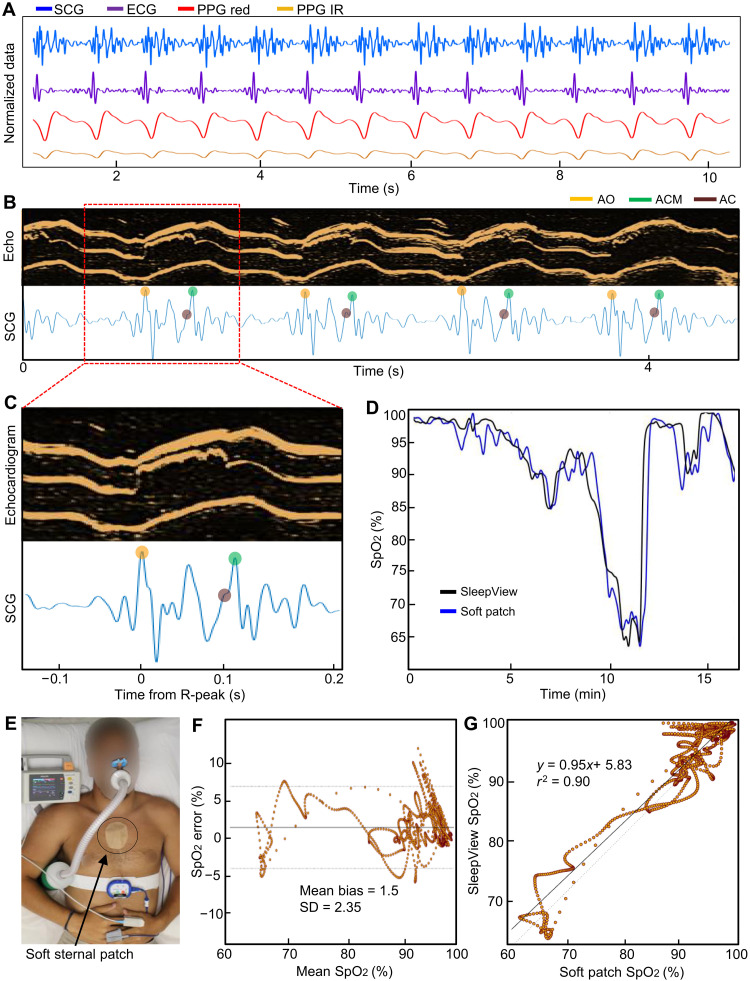
Measured physiological data on the sternum with a soft sternal biopatch with multifunctional sensors. (**A**) Simultaneous recording of SCG, ECG, and PPG signals measured by an all-in-one, wireless soft sternal patch. (**B**) Side-to-side comparison of echocardiogram (echo) and SCG data, with an m-mode view of the parasternal long axis captured to identify aortic valve opening (AO; orange dot), ACM (green dot), and AC fiducials (AC; brown dot). (**C**) Magnified view of a single SCG beat. The fiducials clearly correlate well with the opening of the aortic valve in the echocardiogram recording. (**D**) Simultaneous SpO_2_ recording and comparison between the soft sternal patch and a commercial device (SleepView) during a controlled desaturation induced by a simulator. (**E**) Image of a subject during the controlled desaturation experiment described in (D). (**F** and **G**) Second resolution Bland-Altman diagram (F) and correlation plot (G) for the controlled desaturation experiment.

This study with soft sternal biopatches included nine patients, which measured multiple physiological signals. Among them, four patients exhibited apnea symptoms, and these signals were assessed for quality and repeatability. Figure 3 summarizes a representative set of SCG, ECG, and PPG signals. These data, measured by a patch on the sternum, are sampled at 500, 120, and 200 Hz and filtered with a third-order Butterworth band-pass filter to 4 to 24, 0.5 to 50, and 0.3 to 7 Hz for SCG, ECG, and PPG, respectively. The ECG signal is normalized for R-wave detection and demonstrates high repeatability, as shown in Fig. 3A. The sternum-recorded ECG data show precise PQRST components (fig. S10). A real-time recorded video (movie S3) captures how a soft patch can measure multiple wireless signals simultaneously. The dorso-ventricular SCG data contain clear S1 and S2 complexes corresponding to AO and aortic closure (AC), consistent with the previous recordings (*24*). High SCG repeatability was also demonstrated in three dimensions, as summarized in fig. S11. For validation of the signal quality, we compare the recorded SCG with clinical-grade echocardiography m-mode images of the parasternal long axis, as shown in Fig. 3B. The signals are synchronized on the basis of the R peaks in ECG and periodic tapping. The SCG signal is plotted below the echocardiogram data; the AO, AC movement (ACM), and AC fiducials are automatically detected (details of the signal detection in fig. S12). Figure 3C shows a zoomed-in view of a single SCG beat where the fiducials clearly correlate well with the aortic valve’s opening in the echocardiogram recording. Additional echocardiogram images in figs. S13 and S14 provide the data comparison details, showing the detection of the mitral valve opening and closure. In addition, automated detection of PEP and LVET is possible by using the R-peaks in ECG and the SCG aortic valve opening and closing (fig. S15). In this study, the SCG signal is critical to assessing acute cardiovascular and hemodynamic responses to disturbed breathing. An SCG autocorrelation method could offer a quantitative signal quality evaluation metric by identifying zero, one, and two beat lags, as demonstrated in fig. S16. SpO_2_ is another crucial parameter of interest that sleep clinicians use to detect apneas and hypopneas and characterize overall patient health, and accurate SpO_2_ recording depends heavily on PPG signal quality. To validate the SpO_2_ measurement, SpO_2_ was simultaneously recorded by the soft sternal patch and the FDA-cleared CleveMed SleepView finger PPG probe during controlled desaturations induced by a simulator (AltoLab Altitude Simulator). A time-series graph of the experimental results is shown in Fig. 3D, and the experimental setup is shown in Fig. 3E. It is clear from Fig. 3E that the soft device’s SpO_2_ is highly accurate when compared to SleepView for desaturations of up to 35%, which is beyond the 30% required by the FDA in ISO-80601 for PPG probes. These results are further described in the Bland-Altman diagram in Fig. 3F and the correlation plot in Fig. 3G. Specifically, the slope of 0.95 and *r*^2^ of 0.9 indicate a very high data correlation.

### Experimental findings from overnight, at-home sleep recordings and breathing exercises with symptomatic and control subjects

**Fig. 4.**
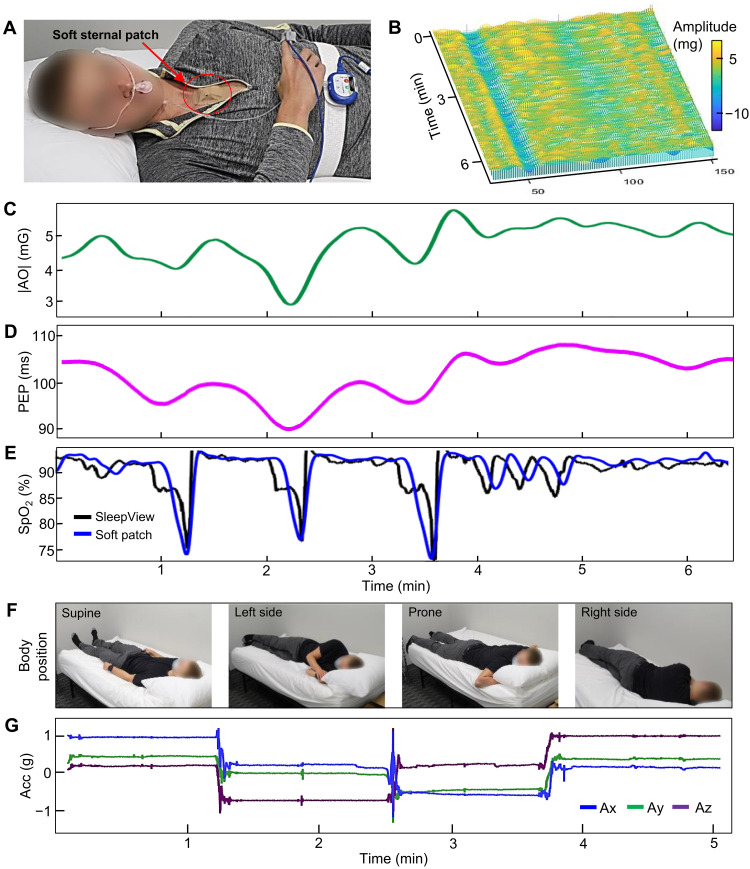
Controlled study and breathing exercises to detect acute hemodynamic changes and subject orientation. (**A**) Photo of a subject wearing an all-in-one, wireless soft sternal patch (sternum) and a commercial device (SleepView on nose, finger, and chest) during sleep. The commercial system is used to validate the performance of the soft patch. (**B**) Waterfall plot of segmented SCG beats during simulated central apneas. Each beat is plotted from left to right. (**C** to **E**) Plots of AO magnitudes (C), PEP data (D), and SpO_2_ data during long and short breath holds (E). The simultaneously recorded SpO_2_ between the soft patch and commercial device shows an excellent agreement. (**F**) Images of subject’s sleeping body positions, including in supine, on the left side, in prone, and on the right side during the study. (**G**) Summarized three-axis acceleration (acc) data that clearly capture the changes of body positions.

Figure 4A shows a photo of a subject who wears an all-in-one soft patch on the sternum along with a commercial, FDA-approved device (SleepView, CleveMed) for simultaneous sleep data recording and producing the SCOPER metrics. Unlike the multisensor-embedded soft patch, the commercial device requires three separate sensors (chest-wrap belt, nasal cannula, and pulse OX on a finger) and a data acquisition system on the chest. We conducted a controlled breathing exercise study with healthy participants to evaluate physiological responses in SCG and PPG signals. Figure 4B shows an overlay of SCG signals during a controlled test segmented by ECG R-peaks. The waterfall plot of segmented SCG beats clearly shows the evolution of fiducial magnitudes with time during simulated central apneas, long and short breath holds. A slice along the AO fiducial plane yields the plot in Fig. 4C, and the delay in the AO from the ECG R-peak is plotted in Fig. 4D. Each signal demonstrates a clear trough after breath-hold termination, indicating acute sympathetic arousal. However, the terse breath-holding elicits no response, meaning that the signals are independent of chest movement or other artifacts related to the breath hold. A plot in Fig. 4E compares the measured SpO_2_ data between the soft patch and SleepView, showing an excellent agreement. The soft patch’s signals respond more rapidly to a desaturation because PPG is recorded in a central location instead of the finger, and the signal here is delayed 10 s to better correlate with a peripheral recording. The details of the SpO_2_ detection algorithm used in this study are summarized in fig. S17. Figure 4F captures a series of photos showing the subject’s body positions, including supine, on the left side, prone, and on the right side during the study. The patch-embedded three-axis acceleration sensor could capture the changes of body positions, as shown in Fig. 4G.

**Fig. 5.**
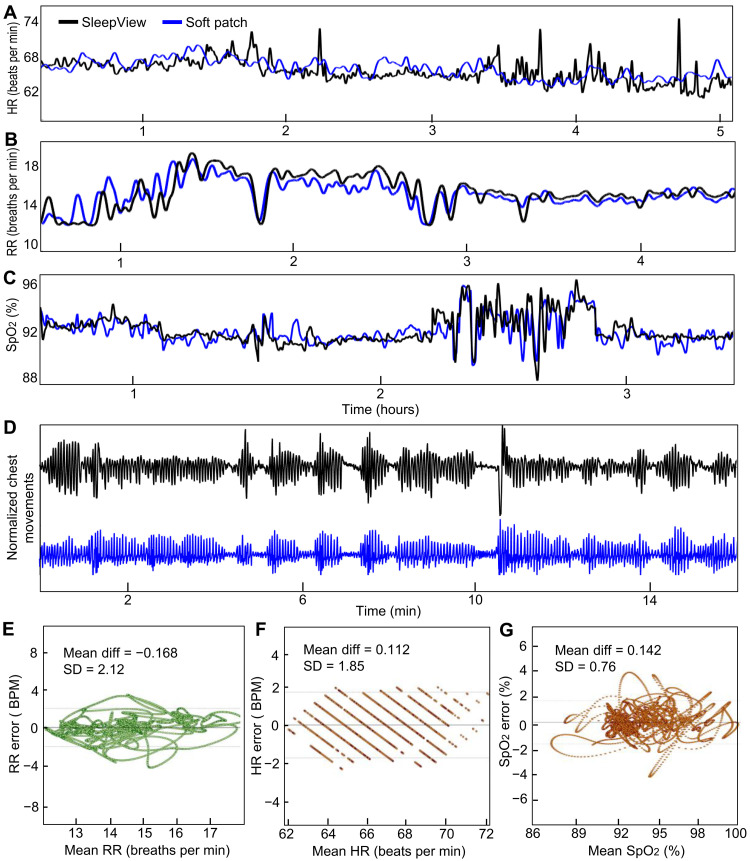
Overnight sleep study with patients and healthy control subjects. (**A**) Comparison of measured HR data over 5 hours between a soft patch and a commercial device, showing a representative dataset from a control subject; the commercial one has sensor delamination during recording. (**B**) Comparison of respiratory rate (RR) data, showing excellent agreement between the two devices. (**C**) Comparison of SpO_2_ data with a patient, showing both apnea events and normal breathing for 3 hours. (**D**) RE data determined from the chest movements of a patient, showing high equivalence with the commercial inductive belt. Unlike the clean data from the soft patch, the SleepView device shows unexpected noise caused by motion artifacts. (**E** to **G**) Bland-Altman diagrams for RR (E), HR (F), and SpO_2_ (G) for three independent nights with the mean difference and SD, indicating highly accurate recordings.

Figure 5 summarizes an overnight sleep study with multiple patients and control subjects. This study aims to assess the wearable device’s efficacy compared to the commercial home sleep monitor SleepView (details of the subject population in table S1). A comparison plot in Fig. 5A shows the simultaneously recorded HR data over 5 hours from the soft patch and SleepView. The result clearly differentiates the signal quality between the two devices, showing smooth curves from the patch and fluctuated irregular peaks from the commercial one. The HR data from SleepView are extracted from the finger-worn, wired PPG sensor, which could be easily dislodged when the subject rolls onto their side. On the other hand, the soft patch’s HR, extracted from ECG data, has negligible effect from motions due to the conformal lamination of the ultrathin device to the skin. There are no external wires, and the dry nanomembrane sensor in the patch maintains intimate contact with the sternum during sleep. Figure 5B shows the comparison of simultaneously recorded respiratory rate (RR) data, showing an excellent agreement. Unlike the HR data, RR signals from the commercial system are calculated from the chest-worn, pressured belt with an accelerometer, which has fewer sensor decoupling issues during sleep. The SpO_2_ measurements shown in Fig. 5C indicate a high correlation between both systems during normal breathing and disturbed breathing during apnea events. Crucially, the soft device maintains PPG signal quality and proper lamination to the sternum during motion induced at apnea termination. The SleepView’s RE data in Fig. 5D are determined by the chest’s tidal movements from the chest-worn belt. In this case, the soft sternal patch shows higher-quality data than the commercial one. The unexpected high-amplitude peaks from the SleepView data would be attributed to motion artifacts and hanging wire movements during a subject’s sleep motion. Three plots in Fig. 5 (E to G) show Bland-Altman diagrams for RR, HR, and SpO_2_ data with the mean difference and SD. The low SD values of around two beats or less for HR and RR and 0.76% for SpO_2_ indicate a high-precision recording of the soft sternal biopatch. These validated metrics are then used as inputs for deep learning networks to detect apnea and hypopnea and classify sleep stages. An example of how these parameters change during an apnea event is shown in fig. S18.

### Sleep analysis and classification of sleep stages with a neural network

**Fig. 6.**
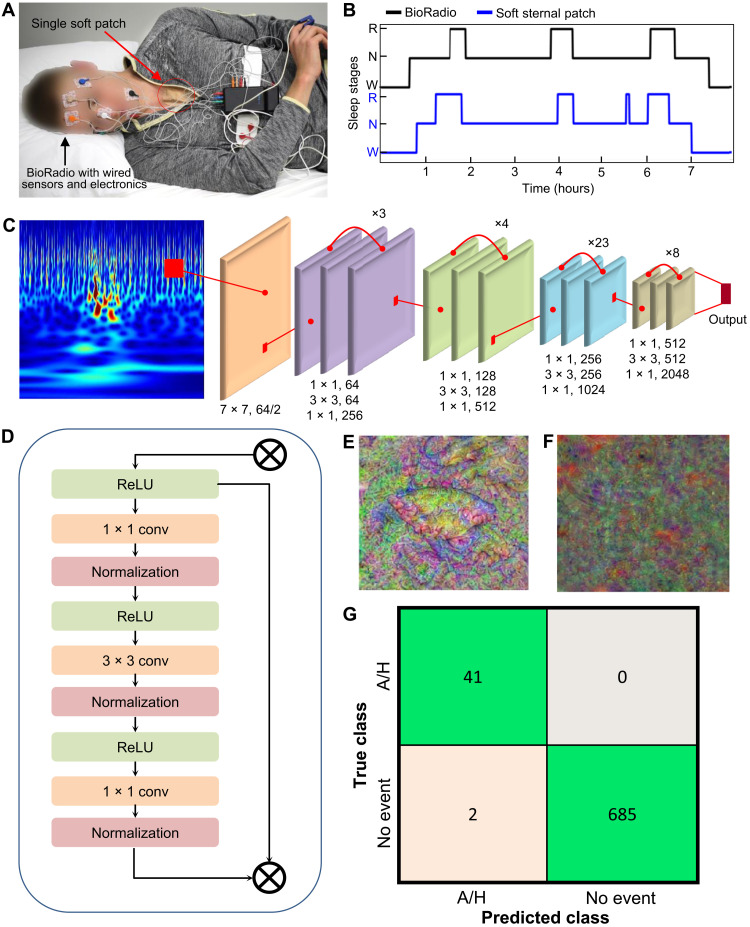
Machine learning implementations for sleep staging and apnea detection. (**A**) Photo of a subject wearing both single soft patch and commercial device (BioRadio) during sleep. The commercial system measures EEG, EOG, and EMG by following the AASM clinical standard. (**B**) Hypnogram comparing the determined sleep stages between the soft patch and BioRadio, showing accurate detection. (**C**) RNN implementation for apnea detection, with an example image of SCG data during an apnea transformed via wavelet element analysis. (**D**) The recurrent node, which forms the building block of the RNN algorithm. (**E** and **F**) Deep activations showing the highly preferred features for apneas (E) and hypopneas (F). (**G**) Confusion matrix demonstrating very high classification accuracy (100% sensitivity and 95% precision) of the soft patch.

Sleep staging was conducted to provide clinicians with critical insight into how a patient’s sleep apnea affects their sleep quality, so more than the standard two-class (sleep versus wake) classification was required. To classify a subject’s sleep into the wake, NREM, and REM stages, An FFNN was trained against sleep stages blind-annotated from electroencephalogram (EEG), electrooculogram (EOG), and electromyogram (EMG) signals collected from the BioRadio in accordance with the AASM clinical guidance (*21*). Stages were annotated into four classes—wake, light sleep, deep sleep, and REM sleep—and the classifier was trained on four classes. However, deep and light sleep were then combined into one class. This was done to avoid a false dilemma problem when differentiating between wake and light sleep, which can cause the classifier to have low confidence during this period of time. Figure 6A depicts the experimental setup. Deep learning was selected for this classification problem because physiological responses to sleep onset and REM sleep involve complicated processes that are best elucidated through advanced feature detection methods (*30*, *31*). Specifically, an FFNN with one hidden layer consisting of 120 neurons was implemented because this model allows for sufficient variable complexity but limits very-high-order interactions that may be subject dependent or a result of overfitting (*31*). Neuron weights and biases were trained with conjugate gradient backpropagation on a set of 262 input features after min-max normalization. Overnight data were collected from six subjects across 10 nights, and two of the subjects exhibited apnea symptoms. The experimental methods are discussed further in Materials and Methods, while the deidentified subject information is provided in table S1. Input features were derived from the set of physiological signals used in the apnea detection algorithm and actigraphy metrics based on the acceleration power spectral density profile. Figure S19 depicts these normalized input features plotted against scoring epochs for a night of sleep. Although the input features show little differentiation with time, the neuron activations in the hidden layer demonstrate sufficient complexity. The average values of each feature and neuron segmented by comparison classification are provided in fig. S20. When validated on a fifth subject, the algorithm demonstrated 82.4% accuracy in three-class classification. A confusion matrix is provided in fig. S21, and a hypnogram comparing three-stage classification with the annotated BioRadio validation set is provided in Fig. 6B. Although sleep staging has been reported in fully wearable sternal patches, very few are able to capture REM sleep, and those that do have reported lower accuracy (*17*, *18*, *32*–*37*). In this work, however, we use the SCG signal to gain valuable insights into the sympathetic innervation of the heart, which allows for greatly improved REM sleep detection. Specifically, PEP, |AO|, and LVET are provided as features in addition to the overall kinetic energy in frequency bands of 5 Hz, ranging from 20 to 40 Hz. This kinetic energy metric is calculated by the magnitude of the Hilbert analytical window of the SCG signal filtered to the specific frequency band of interest, with a window of 5 s and 50% overlap. Unlike traditional wearable alternatives, the soft patch can analyze sleep quality beyond simply wake and sleep, allowing clinicians to differentiate between REM and NREM stages. These results are contextualized with previously reported wearable devices that do not greatly impinge on sleep quality (e.g., EEG caps and facial sensors) in Table 2, which shows a clear increase in classification accuracy that results from improved insight into the sympathetic innervation of the heart that is manifested in the SCG signal. Last, seven total classifiers were attempted to classify sleep stages, including two deep FFNNs, a CNN, a k-nearest neighbor (KNN), support vector machine (SVM), temporal CNN (TCNN), and bagged tree, with the single hidden layer FFNN demonstrating the highest accuracy. The results from these alternative classifiers are provided in fig. S22.

**Table 2.** Sleep stage comparison for wearable and minimally obtrusive devices. Temp, temperature.

**Reference**	**Detectable** signals*	**Device** **description**	**Subjects**	**Wake detection** **accuracy**	**NREM detection** **accuracy**	**REM detection** **accuracy**	**Three-stage** **detection** **accuracy**
This work	ECG, PPG, SCG, and ACC	Soft, wireless sternal patch	6	100%	80.9%	70.4%	82.4%
(*32*)	ECG and ACC	Rigid, wired ECG	32	87.2%	75.5%	88.8%	80.8%
(*18*)	MA and ACC	Tracheal sounds with rigid, wired device	1852	70.0%	85.7%	50.6%	78.3%
(*33*)	ACC and PPG	Rigid, wired ECG	993	86.5	74.1%	75.4%	77%
(*34*)	ACC and PPG	Rigid wristwatch	152	91.5%	65.7%	78.9%	72.9%
(*35*)	ACC and PPG	Rigid wristwatch	60	69.3%	83.4%	71.6%	69%
(*36*)	PPG, ACC, and TEMP	Wearable ring	53	89%	66%	53%	67%
(*37*)	ACC, ECG, and TEMP	Flexible, wireless patch	11	73.3%	59.0%	56.0%	62.1%
(*17*)	MA	Soft, wireless patch	8	72.7%	65.0%	56.3%	56%

*List of detectable signals: ACC, PPG, ECG, RF, SCG, and MM.

### Automated classification of apnea and hypopnea events

Apneas and hypopneas are detected by a deep convolutional network (CNN) with residual connections that is aided by a parametric tree classifier. The deep learning algorithm is provided a continuous wavelet scalogram produced from the SCG signal via element analysis. The SCG signal is extended down in frequency to 0.1 Hz to include respiratory content in addition to the cardiac mechanics associated with valve openings, cardiac kinetics, and turbulent blood flow in the ventricles. The signal is then reconstructed via element analysis and the Morse wavelet described in fig. S23 to create an image capturing only the essential signal components accentuated against the background noise. Input images were derived from 30-s epochs with 20-s overlaps to ensure precision to within 10 s. Features are then learned from this image via a deep learning residual CNN based on the Resnet model. Although the universal approximation theorem provides that an FFNN with a single layer can approximate any function, deeper networks do often perform better in practice because they can learn more complex features and avoid overfitting. However, deep networks are difficult to train because of the vanishing gradient problem, where repeated backpropagation can make gradients rapidly approach zero. In a CNN, each layer contains an input *x_i_* and output *y_i_*, where the input to one layer is the prior’s output (i.e., *x*_*i* + 1_ = *y_i_*). The final output and input are known, and backpropagation is used to create for each layer a mapping H(*x*) such that *y_i_* = H*_i_*(*x_i_*). Solving $\frac{y_{i}}{x_{i}}=H_{i}(x_{i})$ for many connected layers becomes exponentially difficult, so an alternate approximation is introduced. Here, we implement a residual network based on a prior work (*38*), where a residual connection is added such that *y_i_* = H*_i_*(*x_i_*) + *x_i_*, and this architecture is described in fig. S24. In this case, H(*x*) maps the input *x* to the residual ∆*x_i_* = y*_i_* − *x_i_*, and the function $\frac{\text{∆}x_{i}}{x_{i}}=H_{i}(x_{i})$ must be learned. Because this approach has been shown to be suitable with very deep networks, it is implemented here to learn features from the SCG signal (*38*). The overall network architecture with 115 layers and a sample input of a scalogram generated from an apnea event is provided in Fig. 6C. The repeated block architecture is illustrated in Fig. 6D. To better understand the neural network, a random noise image was provided, and the neural network generated output images through inception mediated by gradient ascent. These output images are provided in Fig. 6 (E and F) and show that the apnea detector is looking for high curl patterns demonstrating abnormal breathing in addition to jagged heartbeat signals, whereas the normal breathing detector is expecting highly normal and smooth patterns. The clear difference in the two images is strong evidence that the hidden layers in the classifier can well distinguish between apnea and control scalograms; if the output images were highly similar, then it would indicate a failure of the device to distinguish between classes with high confidence.

The input to the RCNN consisted of the scalogram image described above, and the output is a confidence score between 0 and 1 for apnea detection. This score is fed into a parametric classifier trained with additional signal quality metrics and the features derived previously, like PEP, LVET, HR, RR, and SpO_2_. In addition to the RCNN, TCNN, CNN, long short-term memory (LSTM) and SVM classifiers were implemented, and their sensitivities and precisions are provided in fig. S25. Each of the algorithms consisted of 30-s epochs with 20-s overlaps. The RCNN, LSTM, and SVM algorithms were each trained on 145 input features derived from the same features used in the parametric classifier used in the RCNN and CNN. The CNN was trained on data prepared similarly to the RCNN. For the RCNN and CNN, the RUS boosted tree classifier is trained primarily on signal quality metrics during hand-annotated events and designed to reject classifications that occur as the result of systemic noise, such as when a patient rolls from side to side. It is thus assured that sympathetic arousals and disturbed breathing relate to apnea and hypopnea events. Data were collected overnight from nine subjects (five of whom were symptomatic patients), and one symptomatic subject was precluded from training and used as a validation dataset. For the five symptomatic subjects, a total of eight nights were collected. Nonidentifying participant information is provided in table S1. The experimental methods are further elaborated in Materials and Methods. In this experiment, the soft sternal patch system could correctly detect apneas and hypopneas compared to SleepView data scored by professional sleep clinicians with 100% sensitivity and 95% precision. The RCNN demonstrated the highest accuracy of the five models tested, making it the best choice for this classification problem.

## DISCUSSION

This paper reports a fully integrated, wearable patch for at-home use capable of wirelessly monitoring the mechanical, electrical, and optical signals that arise from acute hemodynamic disturbances during sleep apnea and changes in the sleep stage. The soft sternal patch is the first device to demonstrate simultaneous recording of ECG, PPG, and SCG from a single location, enabling accurate derivation of the vital SCOPER metrics recommended by the AASM to diagnose OSA. In addition, this device provides crucial insights into how the cardiovascular system responds to apneas with second-by-second resolution, which is not possible with even the most expensive and sophisticated echocardiograms or alternative cardiovascular mapping systems. There is no currently marketed device capable of analyzing cardiac mechanics with this resolution. Although clinical echocardiography is often used to determine cardiac timing parameters, it must be administered by a trained professional in a single-use setting, which is not suited for continuous monitoring and is also not available for many underserved communities. Here, this study demonstrates a single wearable patch capable of assessing the cardiovascular and hemodynamic response to sleep apnea. This result is achieved through a thorough study of soft hybrid electronics explicitly designed for the transduction of electrical, optical, and mechanical signals from the chest. First, a study of interfacial physics and soft mechanics demonstrates that the ultralow-amplitude sternal PPG signals can be captured by an elastomeric soft circuit. Second, the excellent mechanical coupling between the device and skin enables the high-quality transduction of mechanical vibrations and electrical potentials at each cardiac beat. Third, signal processing methods are implemented to analyze each of these signals in tandem to provide clinicians with unparalleled insight into their patient’s physiological responses to apnea, and these derived metrics are validated in controlled experiments and overnight recordings with healthy participants. Fourth, machine learning classifiers were designed to automatically derive features from these metrics to identify sleep stages and detect apneas and hypopneas. During overnight studies, the soft patch exhibited excellent conformality, with minimal motion artifacts compared to traditional home sleep tests, and excellent wearability, with very minimal subject training and no tests failed because of human error. In these trials, we report apnea and hypopnea detection with 95% precision and 100% sensitivity compared to data professionally scored by licensed clinicians. In addition to accurately detecting apneas, this device provides clinicians with critical insights into how each apnea affects the patient’s cardiac mechanics, blood oxygenation, and sleep quality. One limitation of this study is the small sample size used to assess the machine learning accuracy. We anticipate additional translational studies that can assess the ability of this promising class of conformal skin-like patches to detect sleep apnea and classify sleep stages. Other limitations include the inability to isolate strain from the rigid units in the device. Ongoing efforts focus on addressing these concerns by using hard-soft elastomer interfaces to reduce strain propagation to the PPG unit from the battery. Overall, the integrated electronics presented here may also allow for a portable, wireless, and continuous recording of cardiovascular and neural health states.

## MATERIALS AND METHODS

### Device fabrication and assembly

Microfabrication of the soft sternal patch device and the nanomembrane ECG electrodes was conducted using micro-electromechanical system (MEMS) lithography processes on a polydimethylsiloxane (PDMS)–coated Si wafer and two-stage transfer to an Ecoflex 00-30 elastomer, as described in section S1 and fig. S2. The device consists of two Cu layers connected with etched vias and three PI layers. Integrated circuits are directly solderable to the board.

### FEA—Circuit bending

FEA was performed using commercial software (Abaqus) to enable high flexibility for the circuit. Circuit bending occurred to 180° for a 3-mm bending diameter along the midsection of the circuit. The circuit connection to the underlying PPG unit was simulated to bend 180° for a 1-mm bending diameter. Circuit layers were meshed with S4R elements, while chip components were meshed with C3D8R elements. The following material properties of Young’s modulus (*E*) and Poisson’s ratio (ν) were used for the circuit layers: *E*_PI_ = 2.3 GPa and ν_PI_ = 0.34; *E*_Cu_ = 119 GPa and ν_Cu_ = 0.34.

### FEA—PPG pressure

FEA was performed to simulate PPG displacement due to applied force. The skin components included both a 100-μm-thick epidermis layer and a 2-mm-thick dermis layer. A PPG unit was partially embedded into a 2-mm-thick elastomer layer. Contact conditions were defined between the PPG unit and skin and the elastomer and skin. To replicate experimental conditions, a linearly increasing displacement was applied to the top surface of the elastomer layer, while the bottom dermis surface was fixed. The elastomer and skin layers were meshed using C3D8R elements. The following material properties of Young’s modulus (*E*) and Poisson’s ratio (ν) were used for the model: *E*_elastomer_ = 1 MPa and ν_elastomer_ = 0.49; *E*_epidermis_ = 1 MPa and ν_epidermis_ = 0.48; *E*_dermis_ = 0.2 MPa and ν_dermis_ = 0.48.

### Skin model experiment—PPG pressure

A skin replica to model the epidermis and dermis layers of human skin was fabricated by first spin coating a 100-μm-thick layer of 10:1 PDMS (Sylgard 184) in a flat dish. This layer was cured at 80°C for 1 hour before spin coating a 2-mm-thick layer of 40:1 PDMS on top of it. The second layer was cured under the same conditions. The 10:1 PDMS layer simulated the epidermis layer, while the 40:1 PDMS layer simulated the softer dermis layer. A circuit with the PPG unit was laminated on top of the skin replica, and a motorized test stand (Mark-10) applied a constant displacement to the top of the circuit. Glass slides were mounted below the skin replica and above the circuit to allow for uniform compression. A force gauge continuously monitored applied force as displacement occurred. To monitor PPG unit displacement, a video camera recorded the motion of the PPG unit into the skin replica. An image processing program (ImageJ) was applied to measure the relative displacement of the PPG unit with respect to the surface of the skin replica at individual video frames.

### Cyclic bending experiment

The device reliability was assessed during cyclic bending to 180° at a radius of 3 mm over 100 cycles. Bending was conducted via the MARK-10 ESM303 motorized force measurement stand. The device was placed on two glass slides and bent down the interface between each. Performance was assessed by Bluetooth transmission of acceleration signals.

### Data collection

Data were collected from human subjects simultaneously with the CleveMed SleepView and BioRadio. The SleepView contains a nasal cannula for airflow measurement, a finger oximeter for HR and SpO_2_, and a respiratory belt for RE. The BioRadio measured chin EMG, EOG for both eyes, and EEG. For the controlled experiments, long-duration breath holds were 40 s to 1 min, and short-duration breath holds were 10 s. Overnight trials occurred in the subject’s home, and each subject set up the device by themselves after training. Exact device placement on the sternum was not mandatory because device placement was not observed to have a large effect on signal quality. All subjects provided informed consent before participation.

### Power management

Power is provided by a battery (150 mAh LiPo, 3.7 V) and regulated to 3.3 V by a TPS63001 voltage regulator and 1.8 V by a TPS62746 voltage regulator. All components except the MAX30102 PPG sensor use 1.8 V, and 3.3 V is required only for the PPG light-emitting diodes. The battery life was more than 10 hours, indicating a power draw of 15 mA. The battery was recharged before each study.

### Serial communications

The nRF52832 BLE SoC communicates with the MAX30102 PPG sensor and MAX30003 ECG front-end circuitry via I2C and the ADXL355 via SPI.

### Sensing circuitry

The ADXL355 is sampled at 500 Hz after a low pass filter (LPF) set at 125 Hz (−3 dB), the MAX30003 is sampled at 128 Hz with an LPF of 61 Hz, and the MAX30102 is sampled at 200 Hz with a proprietary filter. The MAX30102 is oversampled by 4×, so 50-Hz signals are broadcast. In addition, the MAX30102 incorporates proprietary ambient light cancelation through track/hold circuitry. The ADXL is set to 20-bit resolution with range of ±2 g, the MAX30003 is set to 18-bit resolution with range of 65 mVpp, and the MAX30102 is set to 18-bit resolution with a pulse width of 411 μs and 8-μA full-scale range.

### Bluetooth Low Energy (BLE) communications

Data are transmitted via BLE in one service consisting of two characteristics. Because time alignment between the ECG and SCG is of paramount importance, they are combined into a single characteristic. The PPG signal comprises the other characteristic. The ECG/gross acceleration (ACC) and PPG characteristics are broadcast with 240-byte buffers, respectively.

### Android app

The Android tablet receives BLE transmissions from the soft sternal patch device via a custom-designed GATT client app. Data are converted from binary to double, assigned a timestamp, and automatically saved in a CSV file in real time. In addition, data are plotted in real time so that the user can verify function. Because data are saved instantaneously, there is very low risk of data loss. Upon trial completion, the CSV may be emailed to a user from inside the app.

### Signal processing and data analysis

Signal processing and data analysis were conducted in the MATLAB (R2020b) programming language. Real-time data display and processing on the tablet were implemented in Kotlin. Firmware was executed in embedded C.

### Raw data assessment

Signals were assessed in two ways. First, the periodicity was defined as the ratio of an autocorrelation peak for a subsequent beat to the primary peak. Second, signals were ensembled on the basis of the ECG R-wave, dynamically time-warped, and compared to an average beat for a section of 1 min. The greater the average distance from this bear, the less repeatable the signal.

### Apnea and hypopnea detection

Both apnea and hypopnea events were detected without distinction. No two apneas could occur within 10 s based on the AASM guidelines. Should an apnea and hypopnea occur simultaneously, as if often the case, only one event would be reported.

### Sleep apnea algorithm

Overnight data were simultaneously recorded with the FDA-cleared CleveMed SleepView system and the soft sternal patch. Data were collected overnight from five symptomatic subjects and four controls, as summarized in table S1. Among the five symptomatic subjects, two nights were recorded for subjects 1, 3, and 5, totaling in eight nights of symptomatic data. Subjects 4 and 5 also slept with the BioRadio PSG system. Although these data were not included in the PSG scoring, all data recordings were completed for greater than 6 hours, and no more than 15% of data was automatically precluded by signal processing techniques to detect degradation in signal quality. In one case, a trial was repeated after user error in setup. The data were portioned using fourfold cross-validation into equal segments and trained over 100 epochs. Subject 2 was held entirely out of training and validation, and this unseen subject was used to test the models. Subject 2 was selected for validation because they were the only subject that was neither tested twice nor asked to wear the BioRadio system. Professionally licensed sleep clinicians scored training data for the detection of apnea.

### Sleep staging algorithm

Overnight data were simultaneously recorded with the soft patch and the BioRadio system configured to collect EEG, EOG, and EMG signals. An example of the BioRadio configuration is shown in Fig. 6A. Data were collected overnight from six subjects across 10 nights, with two subjects exhibiting apnea symptoms. Of the subjects tested with apnea symptoms, only subjects 4 and 5 wore the BioRadio comparison device in addition to the SleepView. Thus, only these two subjects were included in the sleep staging algorithm development. Professionally licensed sleep clinicians scored sleep stages in 30-s epochs. For the 8910 epochs, 21% were wake time, 63% were NREM sleep, and 16% were REM sleep. NREM was further scored into deep NREM and light NREM, although the final classification problem involved only NREM, REM, and wake classes. Data were input into several machine learning algorithms, as shown in fig. S23, and a single-layer FFNN was chosen. The input classes consisted of 120 derived metrics from the SCG, PPG, and ECG data. HR, HRV, RR, RE, |AO|, LVET, PEP, cardiac kinetic energy, and actigraphy were included. The details of these derived metrics are discussed previously. None of the derived input features were rejected from the classifier. The data were portioned using fourfold cross-validation into equal segments and trained over 500 epochs. Subject 8 was held entirely out of training and validation, and this unseen subject was used to test the models. Subject 8 was selected for the sleep classification validation because they were the only subject that was only tested once and demonstrated the evenest distribution of sleep stages without imbalances.

### Bending stiffness calculation

The device bending stiffness depends primarily on the deflection of the Cu and PI thin-film depositions (assuming that the bending is not along an axis containing a rigid integrated circuit). This bending can be analytically modeled as a composite beam with one end fixed and assuming a homogenous film. If force is applied on the circuit by an unfirmly distributed load *q*, then the bending stiffness is determined to be

$$k=\frac{\mathrm{qL}}{\delta }=\frac{8\mathrm{EI}}{L^{3}}$$

where *k* is the bending stiffness, *L* is the circuit length, δ is the beam deflection at its tip, *E* is the Young’s modulus, and *I* is the moment of inertia. Because the beam is a composite of five layers (fig. S2), the transformed-section method is implemented to transform the cross section of the beam unto an equivalent mechanical model composed of a uniform material. In this case, the width (*w*) of each layer is dependent on the ratio of Young’s moduli

$$w_{2}=\frac{E_{2}}{E_{1}}w_{1}$$

The moment of inertia for this transformed cross section is determined from the parallel-axis theorem to be

$$I_{\text{total}}=\sum (I_{i}+A_{i}d_{i}^{2})$$

where

$${\bar{I}}_{i}=\frac{1}{12}\frac{E_{i}}{E_{\text{PI}}}w_{i}h_{i}^{3}$$

$$A_{i}=\frac{E_{i}}{E_{\text{PI}}}w_{i}h_{i}$$

$$d_{i}=|y_{i}-\bar{y}|$$

thus

$$I_{\text{total}}=\sum \frac{E_{i}}{E_{\text{PI}}}w_{i}h_{i}(\frac{1}{12}h_{i}^{2}+|y_{i}-\bar{y}|)$$

where *I*_total_ and *I_i_* are the moments of inertia for the cross section and individual layers, respectively; *A_i_* is the area of each layer; *d_i_* is the vertical distance from the neutral axis; *h_i_* is the layer thickness; *y_i_* is the centroid height; and $\bar{y}$ is the neutral axis height. Substituting the moment of inertia equation yields

$$I_{\text{total}}=\frac{8E_{\text{PI}}I}{L^{3}}\sum \frac{E_{i}}{E_{\text{PI}}}w_{i}h_{i}(\frac{1}{12}h_{i}^{2}+|y_{i}-\bar{y}|)$$

The circuit is composed of three PI layers and two Cu layers as illustrated in fig. S2. The thickness of each layer is 2.6, 1.5, 8.7, 1.5, and 2.8 μm for the bottom PI, bottom Cu, middle PI, top Cu, and top PI layers, respectively. *E*_PI_ and *E*_Cu_ are 2.5 and 119 GPa, respectively. The width of the circuit is 22 mm, and the length is 43 mm. Solving with these parameters, the resulting bending stiffness is 0.00658 N/m.

### Continuous wavelet transform via element analysis

Signals can be transformed with Morse functions ψ_μ, γ_ with order μ and family γ to obtain information about events that are obscured in noise. Here, we represent a signal *x*(*t*) by a Morse wavelet element function

$$x(t)=\sum_{n=1}^{n}R\{c_{n}\psi_{\mu ,\gamma }(\frac{t-t_{n}}{\rho_{n}})\}+x_{e}(t)$$

where the complex parameter *c_n_* = ∣*c_n_*∣e^iϕ*_n_*^ sets the amplitude ∣*c_n_*∣ and phase ϕ*_n_* of the event *t_n_*, and ρ*_n_* sets the event scale. *x*_e_(*t*) is the signal error function. The resultant transform is described by the parameters ∣*c_n_*|, ϕ*_n_ t_n_*, ρ*_n_*. Taking the wavelet transform of *x*(*t*) using a (β,γ) Morse wavelet ψ_β, γ_ yields

$$x(t)=\frac{1}{2}\sum_{n=1}^{n}\int_{-\infty }^{\infty }\psi_{\beta ,\gamma }^{*}(\frac{t-\tau }{s})\psi_{\mu ,\gamma }(\frac{t-t_{n}}{s})\mathrm{dt}+\varepsilon_{\beta ,\gamma }(\tau ,s)$$

where ε_β, γ_(τ, *s*) is the wavelet transform of the noise signal, and τ and *s* represent the transformed time and scales, respectively. The transform maxima occur at the time/scale locations $\left(\hat{\tau },\hat{s}\right)$ for which the wavelet transform modulus *w*_β, γ_ is at a local maximum or formally

$$\frac{\partial^{2}}{\partial \tau^{2}}|w_{\beta ,\gamma }(\tau ,s)|=\frac{\partial^{2}}{\partial s^{2}}|w_{\beta ,\gamma }(\tau ,s)|$$

The wavelet transform at the maxima points can be determined by the function

$$\zeta_{\left(\beta ,\mu ,\gamma \right)}(\tau ,s)=\frac{\alpha_{\beta ,\gamma }\alpha_{\mu ,\gamma }}{\alpha_{\beta +\mu ,\gamma }}\frac{s^{\beta }}{{\left(\sqrt[{\gamma }]{s^{\gamma }+1}\right)}^{\beta +\mu +1}}\psi_{\beta +\mu ,\gamma }(\frac{\tau }{\sqrt[{\gamma }]{s^{\gamma }+1}})$$

where the normalizing constant $\alpha_{\beta ,\gamma }=2{\left(\frac{e\gamma }{\beta }\right)}^{\beta /\gamma }$, by the expression

$$w_{\beta ,\gamma }(\tau ,s)=\frac{1}{2}\sum_{n=1}^{N}c_{n}\zeta_{\left(\beta ,\mu ,\gamma \right)}(\frac{t-t_{n}}{s},\frac{s}{\rho_{n}})$$

for *N* events. The parameters ∣*c_n_*|, ϕ*_n_ t_n_*, ρ*_n_* may be inferred from the observation that for each *n*th event, the maximum will occur at time *t_n_* and scale *s_n_* such that

$$w_{\beta ,\gamma }(t_{n},s_{n})=\frac{1}{2}c_{n}\zeta_{\left(\beta ,\mu ,\gamma \right)}^{\text{max}}$$

where the maximum of the modified wavelet ζ_(β, μ, γ)_ is given as

$$\zeta_{\left(\beta ,\mu ,\gamma \right)}^{\text{max}}=\frac{\alpha_{\beta ,\gamma }\alpha_{\mu ,\gamma }}{2\pi \gamma }\Gamma (\frac{\beta +\mu +1}{\gamma })\frac{{\left(\frac{\beta }{\mu +1}\right)}^{\frac{\beta }{\gamma }}}{{\left(\frac{\beta }{\mu +1}+1\right)}^{\frac{\beta +\mu +1}{\gamma }}}$$

yielding the transform parameters

$${\hat{t}}_{n}={\hat{\tau }}_{n},{\hat{\rho }}_{n}=,\frac{{\hat{s}}_{n}}{\sqrt[{\gamma }]{\frac{\beta }{\mu +1}}},\text{and}\;{\hat{c}}_{n}=2\frac{w_{\beta ,\gamma }(t_{n},s_{n})}{\zeta_{\left(\beta ,\mu ,\gamma \right)}^{\text{max}}}$$

### In vivo human study

The study involved healthy and symptomatic subjects. For healthy control subjects, the study was conducted by following the Georgia Tech–approved IRB protocol (#H20211). For patients, the study was conducted at Huxley by following the Sterling IRB protocol (#7750-BTorstrick). Before the study, all subjects agreed with the study procedures and provided signed consent forms.

## Supplementary Materials

### This PDF file includes:

Section S1 Figs. S1 to S25 Tables S1 and S2 Legends for movies S1 to S3
