# Supplementary material for: At-home wireless monitoring of acute hemodynamic disturbances to detect sleep apnea and sleep stages via a soft sternal patch
Source: Sci Adv. 2021 Dec 22;7(52):eabl4146. doi: 10.1126/sciadv.abl4146 (PMC8694628; doi:10.1126/sciadv.abl4146)
Supplement: Supplementary file 1 — Section S1 Figs. S1 to S25 Tables S1 and S2 Legends for movies S1 to S3 [file sciadv.abl4146_sm.pdf]

Science Advances
AAAS

Supplementary Materials for

At-home wireless monitoring of acute hemodynamic disturbances to detect sleep apnea and sleep stages via a soft sternal patch

Nathan Zavanelli, Hojoong Kim, Jongsu Kim, Robert Herbert, Musa Mahmood, Yun-Soung Kim, Shinjae Kwon, Nicholas B. Bolus, F. Brennan Torstrick, Christopher S. D. Lee, Woon-Hong Yeo*

*Corresponding author. Email: whyeo@gatech.edu

Published 22 December 2021, *Sci. Adv.* **7**, eabl4146 (2021)
DOI: 10.1126/sciadv.abl4146

The PDF file includes:

Section S1
Figs. S1 to S25
Tables S1 and S2
Legends for movies S1 to S3

Other Supplementary Material for this manuscript includes the following:

Movies S1 to S3

Section S1. Microfabrication of ultrathin metal/polymer composites

a) Stretchable electrode fabrication
    1) Spincoat PDMS at 3000 rpm for 30 sec.
    2) Spincoat 1st polyimide (PI, PI-2610, HD MicroSystems) at 5000 rpm for 1 min.
    3) Cure PI in a vacuum oven at 300 °C for 1 hr.
    4) Deposit 5 nm-thick Cr and 100 nm-thick Au by sputtering.
    5) Spincoat photoresist (PR, Microposit SC1827, MicroChem) at 3000 rpm for 30sec.
    6) Bake PR on a hot plate at 100 °C for 2 min.
    7) Align with a photomask and expose UV light, intensity of 15 mJ/cm2, for 7 sec.
    8) Pattern metal layers with electrode design using photolithography.
    9) Develop exposed PR with a developer (AZ 300MIF, Integrated Micro Materials).
    10) Etch exposed Cr with Cr etchant (Chrome Mask Etchant 9030, Transene).
    11) Etch exposed Au with Au etchant (GE-8110, Transene).
    12) Etch exposed PI using reactive ion etcher (RIE) at 150 W, 150 mTorr, and 20sccm of O2 for 13 min.
    13) Remove residual materials with acetone and foam-tip swabs.

b) Flexible circuit fabrication
    1) Spincoat PDMS at 3000 rpm for 30 sec.
    2) Spincoat 1st PI (PI-2610, HD MicroSystems) at 5000 rpm for 1 min.
    3) Cure PI in a vacuum oven at 300 °C for 1 hr.
    4) Deposit 500 nm-thick Cu by sputtering.
    5) Spincoat PR (Microposit SC1827, MicroChem) at 3000 rpm for 30 sec.
    6) Bake PR on a hot plate at 100 °C for 2 min.
    7) Align with a photomask and expose UV light, intensity of 15 mJ/cm2, for 7 sec.
    8) Develop exposed PR with a developer (AZ 300MIF, Integrated Micro Materials).
    9) Etch exposed Cu with Cu etchant (APS-100, Transene) diluted with DI water(APS-100:DI water=2:1).
    10) Strip PR with acetone and foam swabs, followed by IPA and DI water rinse.
    11) Dehydration on a 100 °C hot plate for 1 min.
    12) Spincoat the first layer of 2nd PI (PI-2524, HD MicroSystems) at 950 rpm for 1 min
    13) Cure PI on a hot plate at 100 °C for 5 min and in a vacuum oven at 250 °C for 3hr including ramping from room temperature.
    14) Spincoat the second layer of 2nd PI at 950 rpm for 1 min.
    15) Cure PI on a hot plate at 100 °C for 5 min and in a vacuum oven at 250 °C for 3hr including ramping from room temperature.
    16) Spincoat PR (AZ P4620, Integrated Micro Materials) at 1000 rpm for 30 sec.
    17) Bake PR on a hot plate at 90 °C for 4 min.
    18) Align with a photomask and expose UV light, intensity of 15 mJ/cm2, for 2 min.
    19) Develop exposed PR with a developer (AZ 400K, Integrated Micro Materials) diluted with DI water (AZ 400K:DI water=1:3).
    20) Etch exposed PI using reactive ion etcher (RIE) at 150 W, 150 mTorr, and 20sccm of O2 for 45 min.
    21) Strip PR with acetone and foam swabs, followed by IPA and DI water rinse.
    22) Deposit 1.5 μm-thick Cu by sputtering.

23) Spincoat PR (AZ P4620) at 2000 rpm for 30 sec.
24) Bake PR on a hot plate at 90 °C for 4 min.
25) Align with a photomask and expose UV light, intensity of 15 mJ/cm2, for 80 sec.
26) Develop exposed PR with a developer (AZ 400K) diluted with DI water (AZ400K:DI water=1:3).
27) Etch exposed Cu with Cu etchant (APS-100) diluted with DI water (APS-100:DI water=2:1).
28) Strip PR with acetone and foam swabs, followed by IPA and DI water rinse.
29) Dehydration on a 100 °C hot plate for 1 min.
30) Spincoat 3rd PI (PI-2524) at 2000 rpm for 1 min.
31) Cure PI on a hot plate at 100 °C for 5 min and in a vacuum oven at 250 °C for 3hr including ramping from room temperature.
32) Spincoat PR (AZ P4620) at 2000 rpm for 30 sec.
33) Bake PR on a hot plate at 90 °C for 4 min.
34) Align with a photomask and expose UV light, intensity of 15 mJ/cm2, for 80 sec.
35) Develop exposed PR with a developer (AZ 400K) diluted with DI water (AZ400K:DI water=1:3).
36) Etch exposed PI using RIE at 150 W, 150 mTorr, and 20 sccm of O2 for 18 min.
37) Strip PR with acetone and foam swabs, followed by IPA and DI water rinse.

c) IC integration
1) Peel off the circuit from the wafer with a water-soluble tape.
2) Transfer the flexible circuit board onto a glass slide and remove water soluble tape with DI water.
3) Use stencil to deposit low-temperature solder paste (alloy of Sn/Bi/Ag (42%/57.6%/0.4%), ChipQuik Inc.) with stainless-steel stencil on the top surface of circuitry.
4) Mount the electronics components.
5) Reflow solder the components on a hot plate by applying heat according to the temperature profile recommended by the solder paste manufacturer.
6) Update the firmware of the Bluetooth-microcontroller.
7) Peel off from the glass slide, and solder LED/photodiode unit on the back side of the circuitry by locally applying heat with hot-air gun.

d) Circuit transfer to elastomer
1) Prepare 7.5 g of Ecoflex Gel (Smooth-On), and 2.5 g of Ecoflex 00-30 (Smooth-On) and mix them together to make 500 µm thick elastomer membrane.
2) Pour the elastomer mixture onto a polystyrene petri dish (FB0875714, Fisher Scientific).
3) Cure the elastomer at room temperature for 5 hr.
4) Cut and peel off the elastomer membrane from the petri dish.
5) Transfer the flexible circuit onto the elastomer membrane.
6) Encapsulate the remaining exposed circuit area with Ecoflex 00-30.

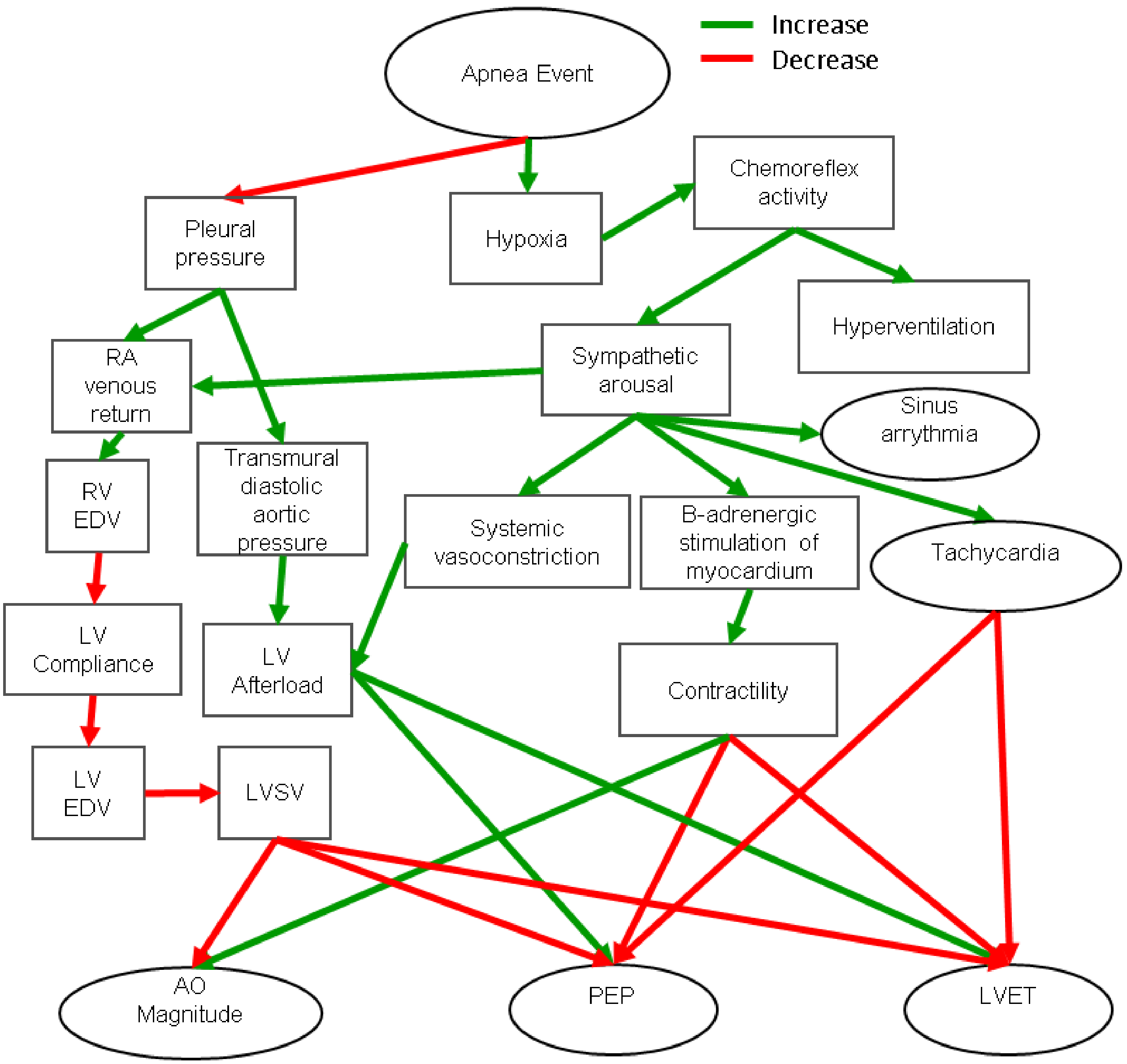

Figure S1. Hypothesized mechanisms for hemodynamic changes during OSA. The following physiological changes are hypothesized to occur in healthy subjects during OSA. These hemodynamic effects are typically hidden in traditional sleep monitoring devices, whereas the proposed patch can measure AO magnitude, PEP, and LVET.

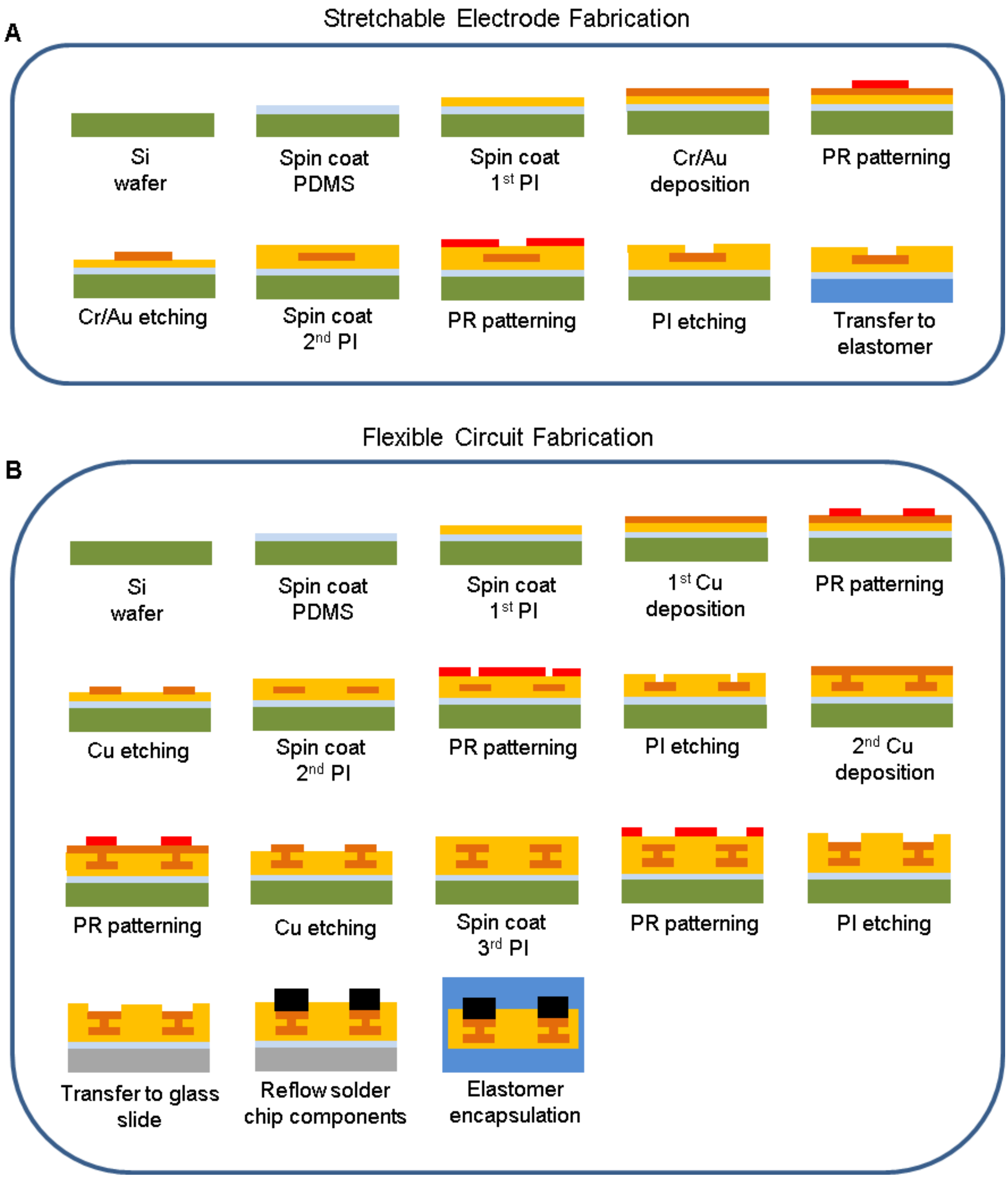

Figure S2. Microfabrication process for flexible electronics and their integration with soft elastomer substrates. (A) Electrode microfabrication and integration. (B) Flexible circuit microfabrication, assembly, and integration for multi-layer electronics.

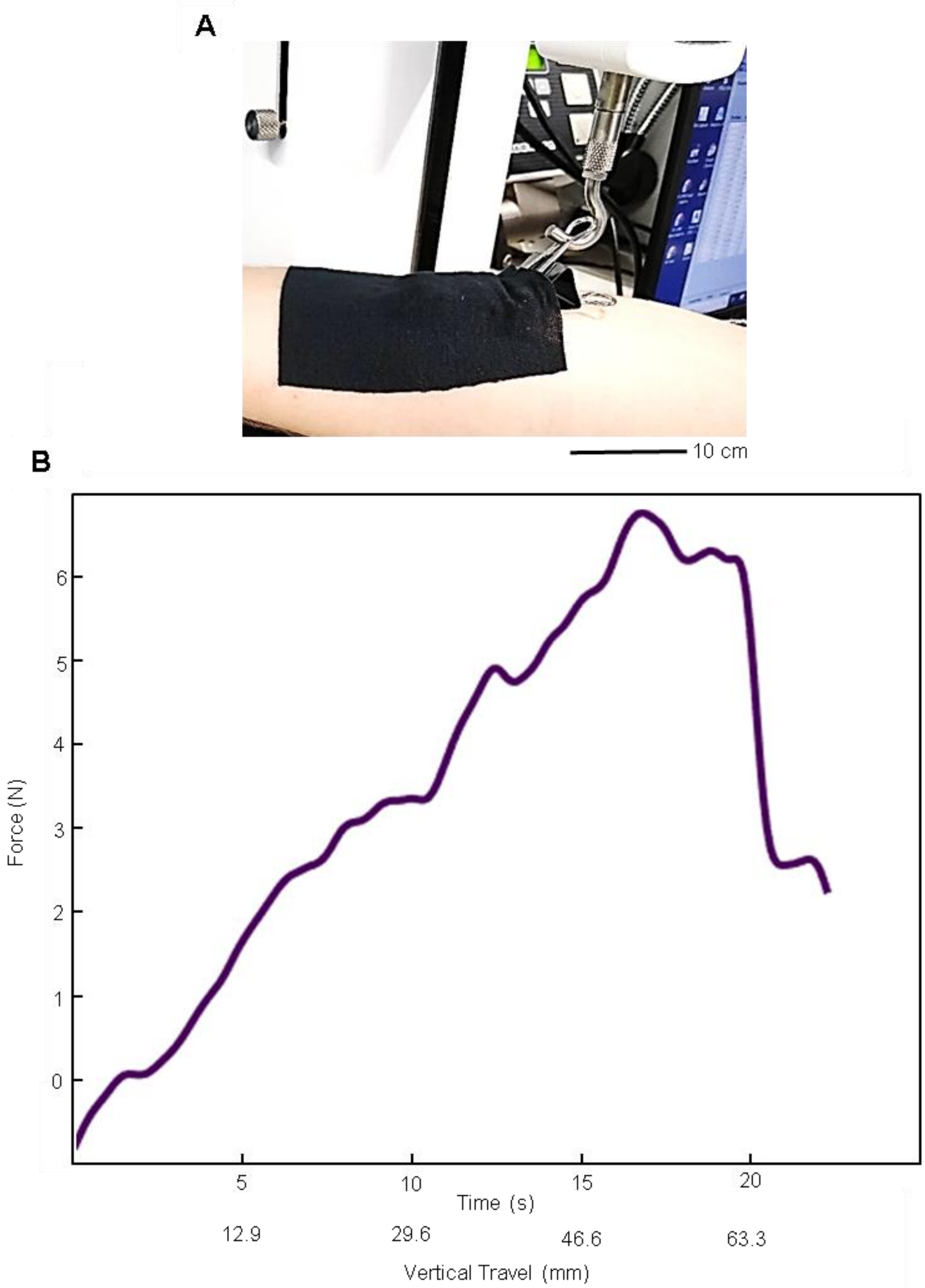

Figure S3. Device adhesion test. (A) Image of the device during delamination from the skin and (B) corresponding delamination force as a function of both time and vertical displacement.

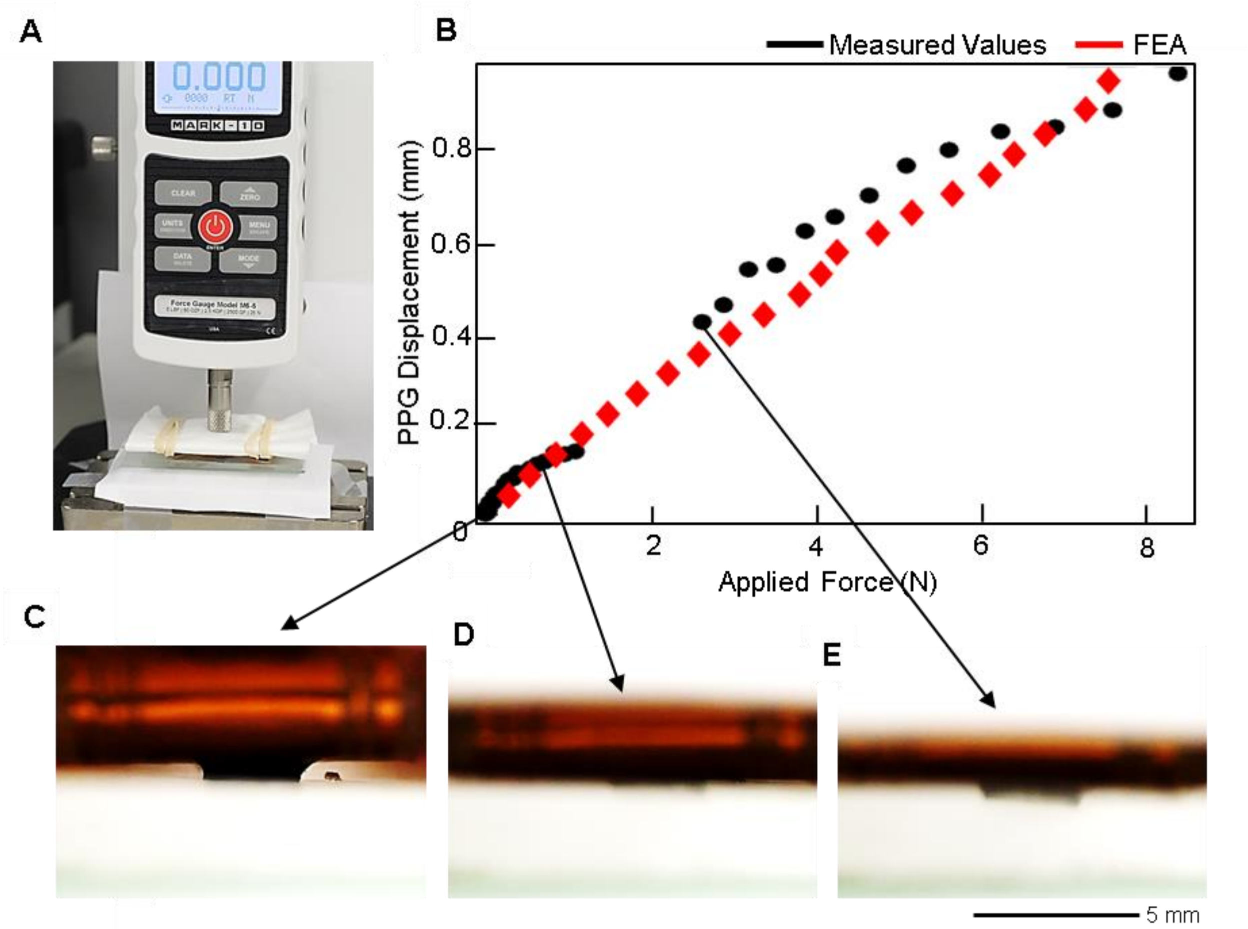

Figure S4. Applied pressure experiment with the soft device. (A) Image of the experimental setup. (B) Graph of PPG displacement into the skin as a function of applied pressure. (C-E) images captured from a video of the device during compression.

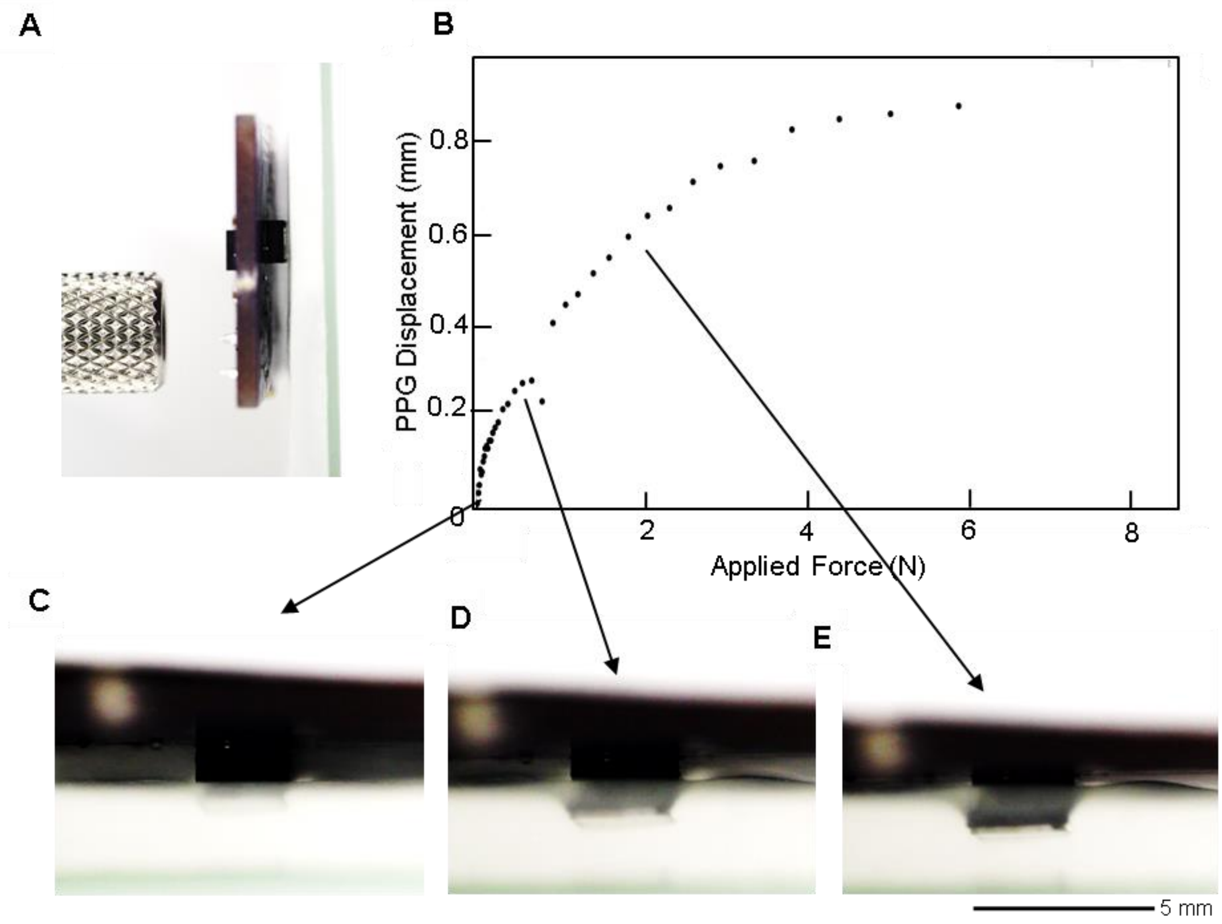

Figure S5. Applied pressure experiment with a rigid board. (A) Image of the rigid board. (B) PPG displacement versus applied force. (C-E) images of the board compressing into the skin mold for various applied pressure regimes.

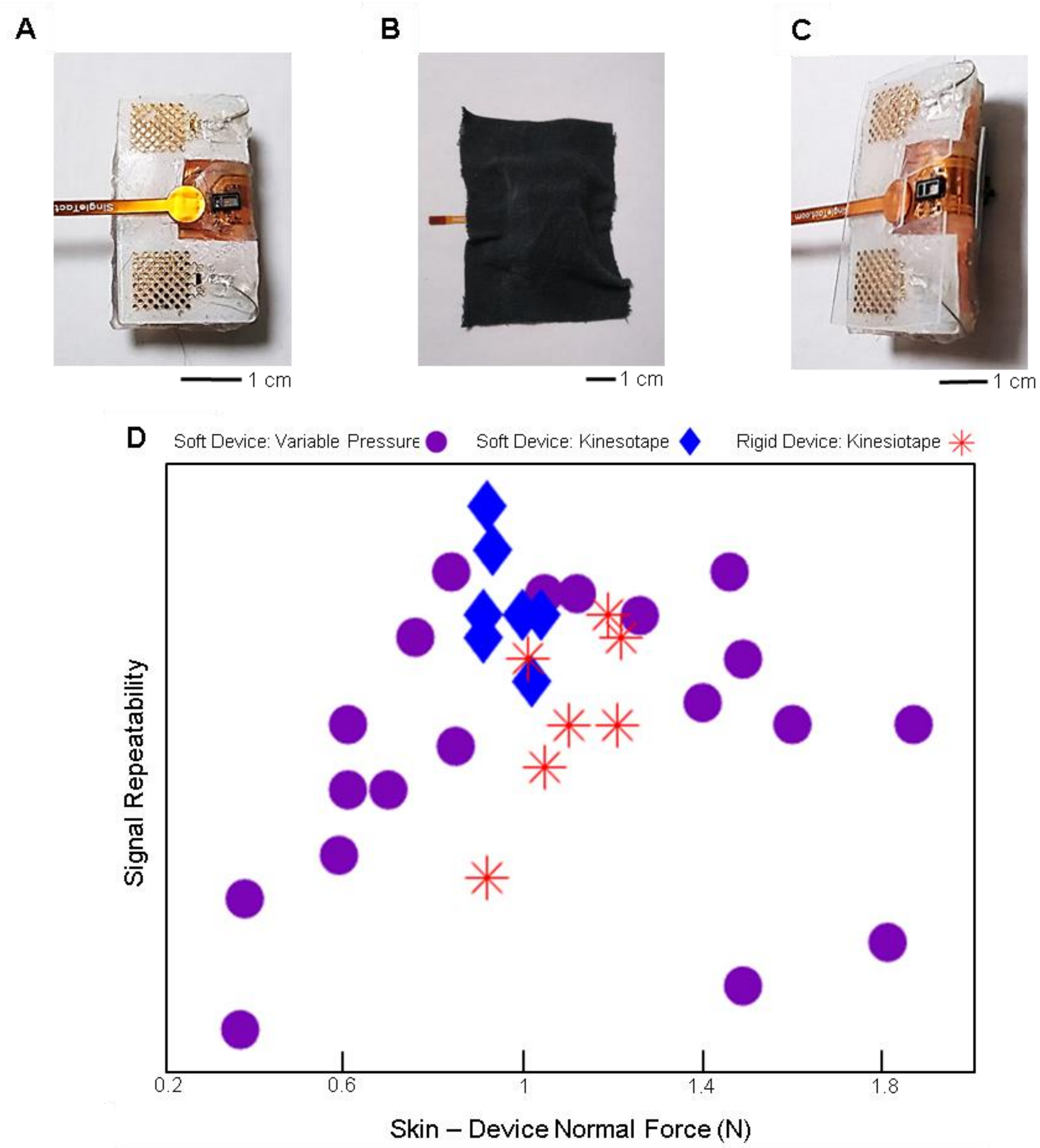

Figure S6. Experiment to determine the optimal contact force. Image of the device with force sensor and (A) no tape to allow pressure variation, (B) Kinesiotape as used during overnight studies, and (C) device with rigid plastic layer and Kinesiotape. (D) Signal repeatability (as a surrogate for SNR) plotted for three experimental conditions across a variety of applied forces between the device and skin.

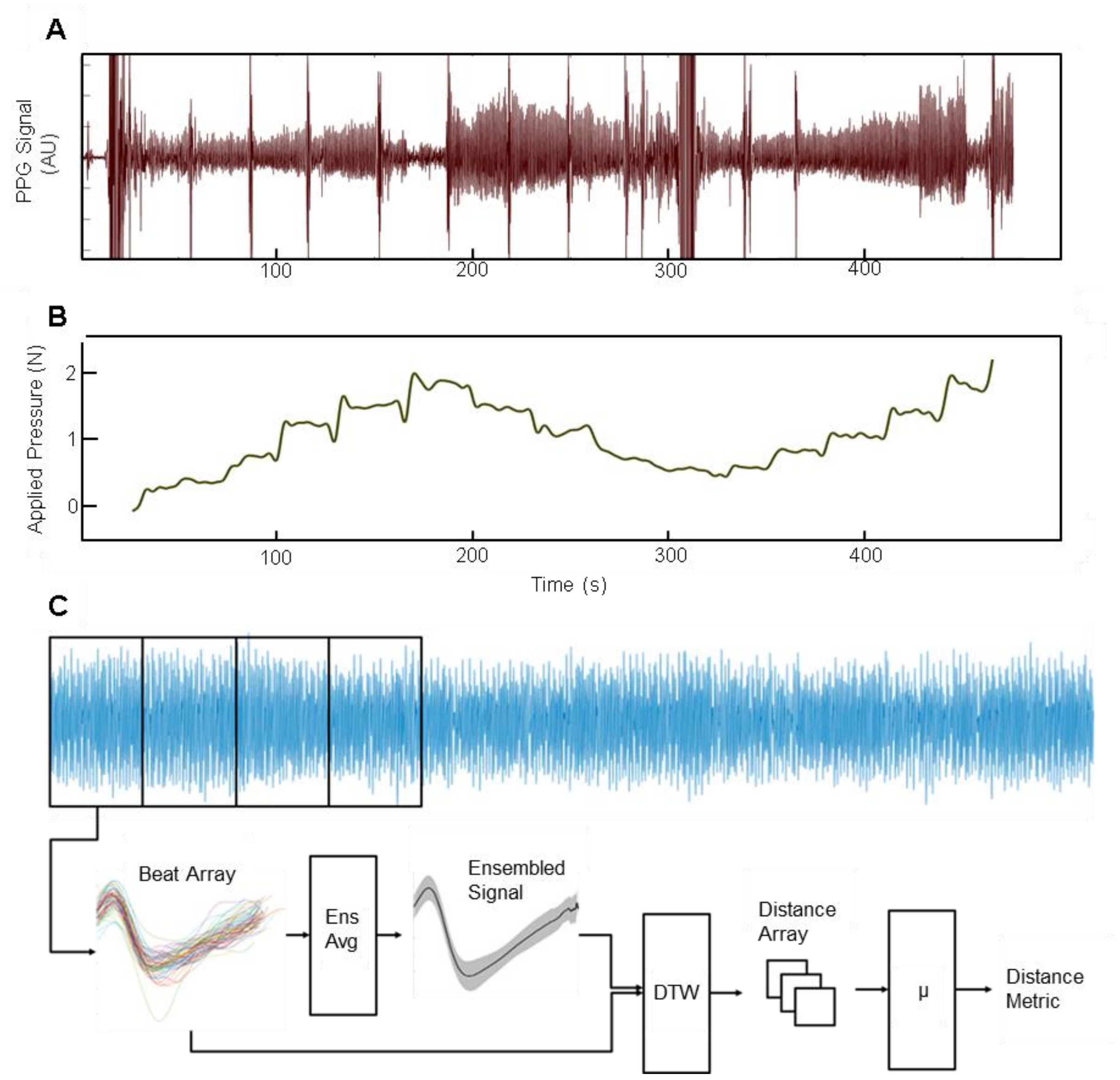

Figure S7. PPG SNR vs. applied force experiment. (A) Filtered PPG signal plotted alongside with (B) applied pressure as a function of time. (C) Signal quality determination through ensembled averaging. This method averages all beats in a segment and averages them, controlling for heart rate. The signal repeatability is captured by the beat standard deviation, and an irregular beat is considered to be noise.

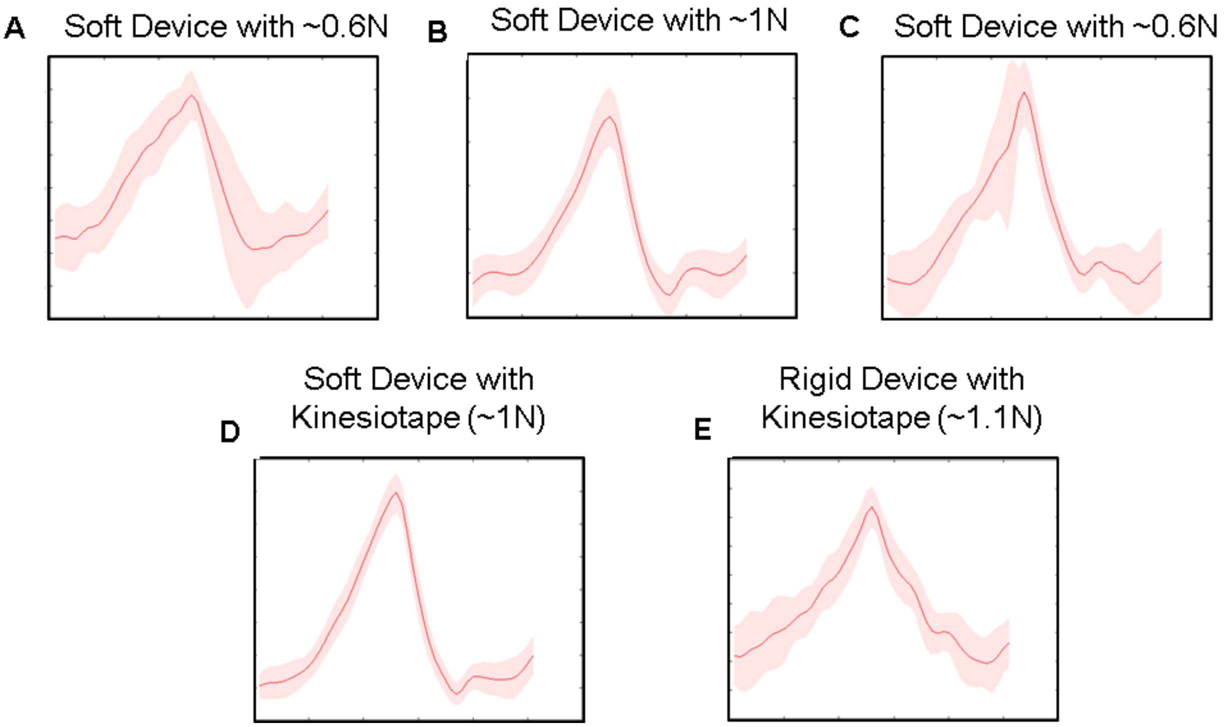

Figure S8. Representative ensembled signals from the applied force experiment. Ensembled signals showing PPG beat average and standard deviation over a 20s interval for the soft device with low (A), medium (B), and high (C) pressure. The experiment was repeated with the final device (d) and compared to the same setup, but with a rigid bottom (E).

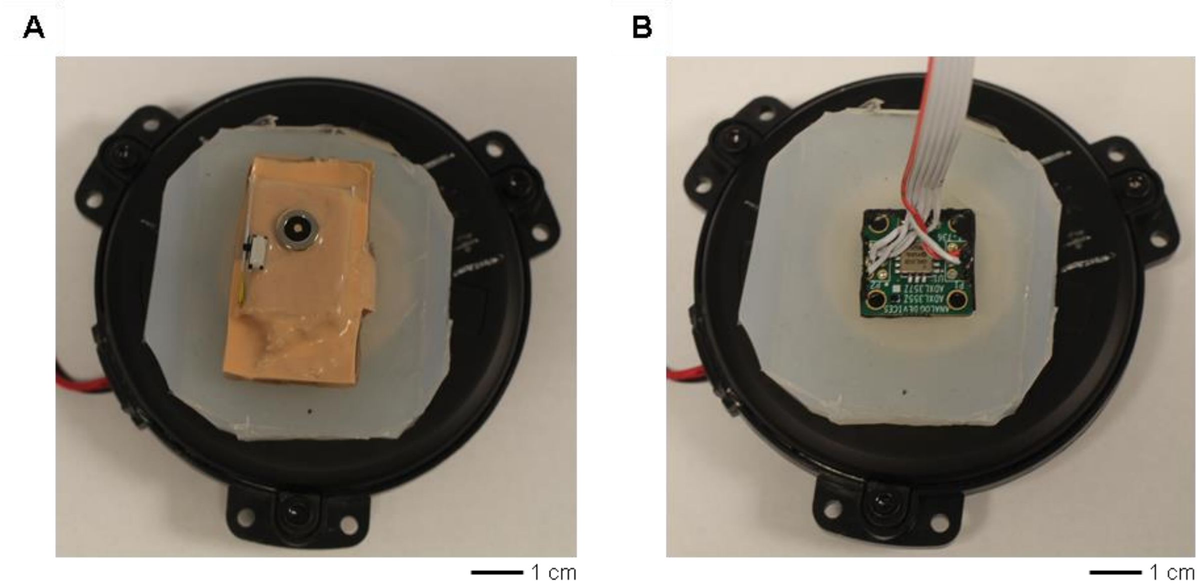

Figure S9. Experimental setup for the skin conformality experiment. (A) Image of the soft sternal patch and (B) image of the rigid comparison device.

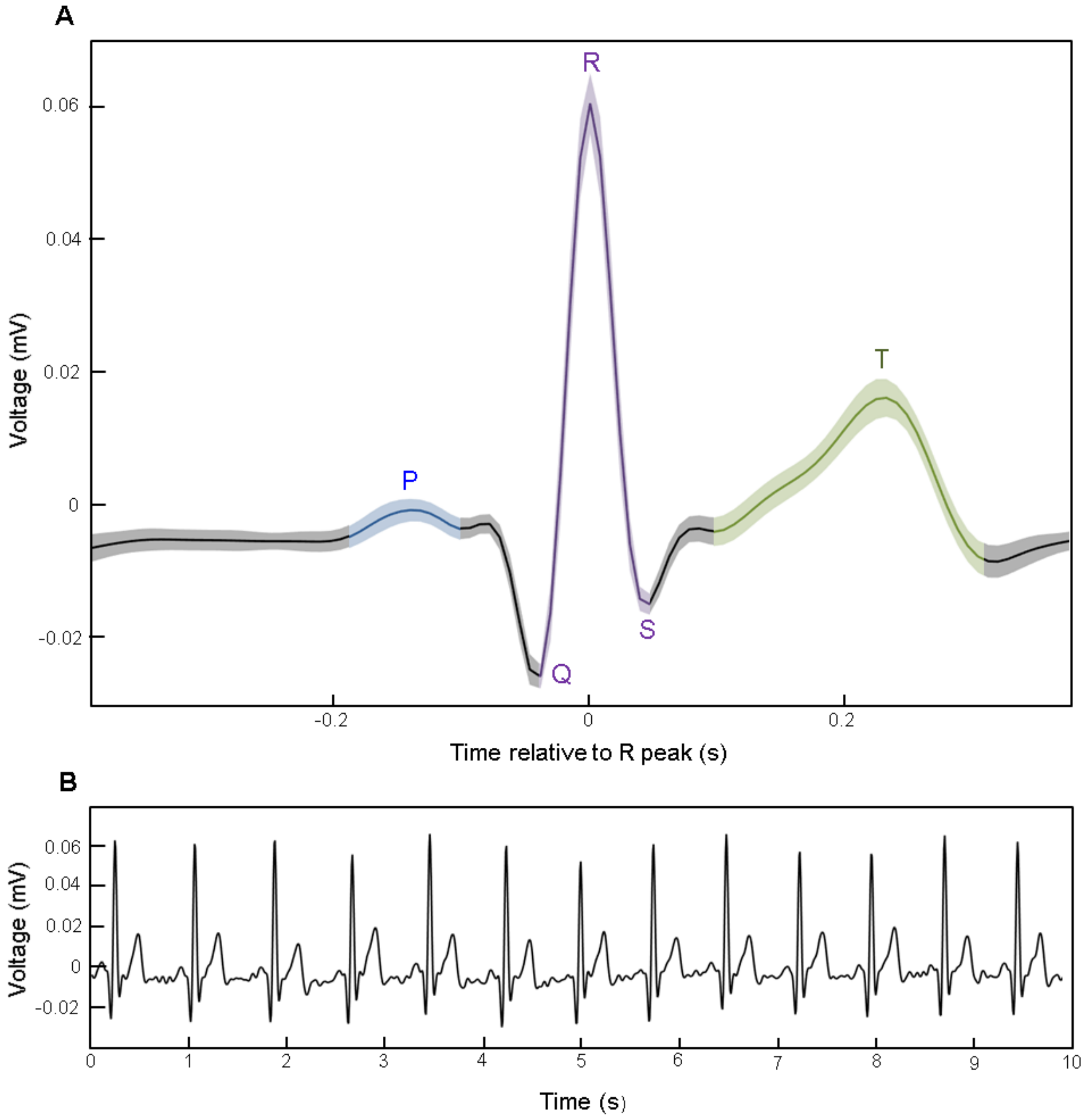

Figure S10. Representative ECG signals. (A) Average ECG signal from an ensemble of 30s with the standard deviation. The P, QRS, and T waves are colored and annotated. (B) ECG signal recording during the first 10s of the data analyzed above.

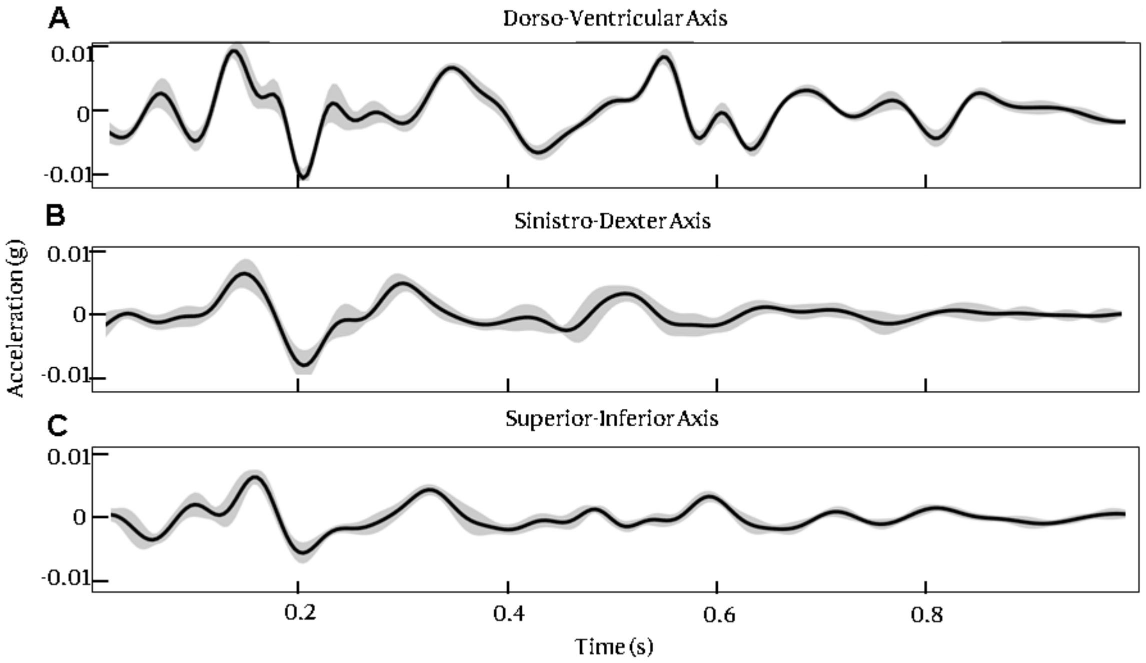

Figure S11. SCG signal in three axes. Simultaneously recorded ensemble-averaged SCG signals in the (A) dorso-ventricular (Az), (B) sinistro-dexter (Ax), and (C) superior-inferior (Ay) axes. The dark line marks the signal mean and the translucent are two standard deviations.

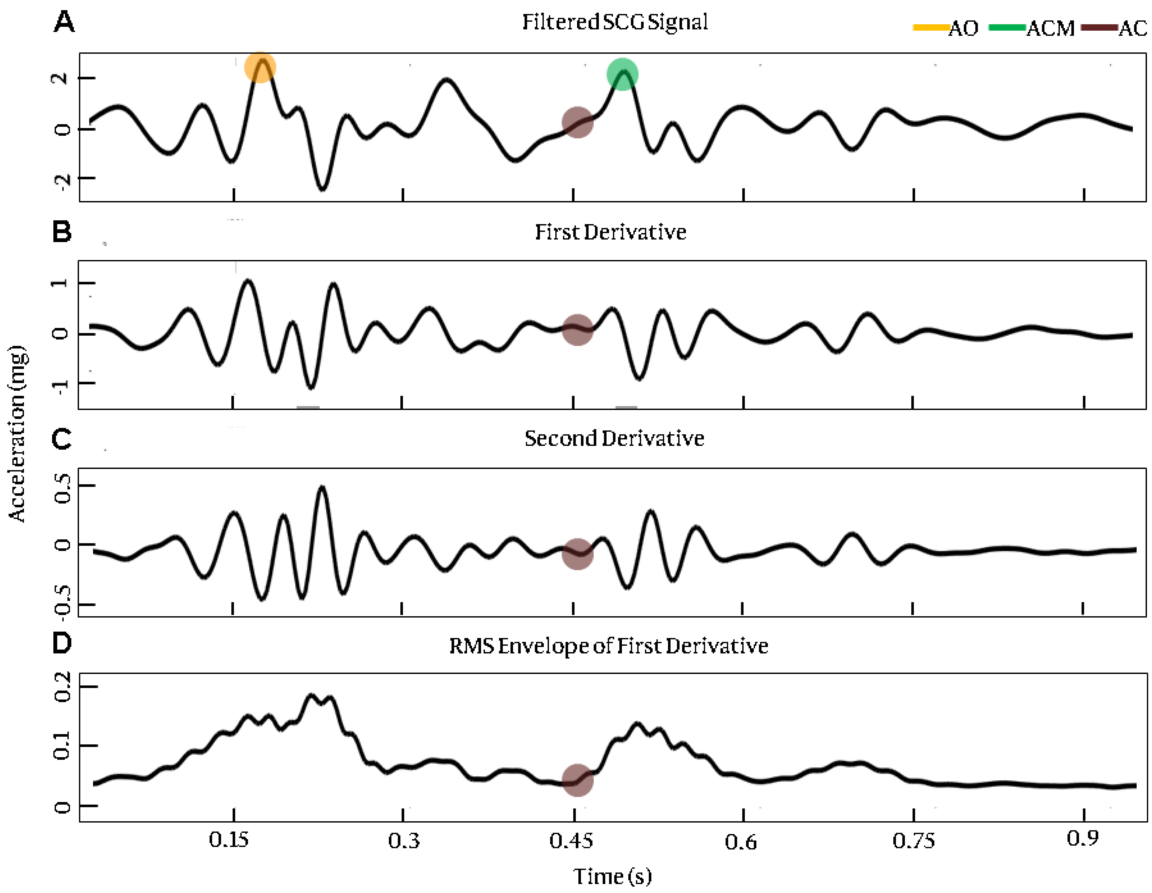

Figure S12. Detection of aortic closure from SCG. The aortic closure point detected in the (A) filtered SCG signal, the (B) first derivative, (C) second derivative and (D) RMS envelope of first derivative. Note that the AO point is poorly defined in the filtered signal but is prominent as the point where the derivative RMS content first increases after a steady period during systole.

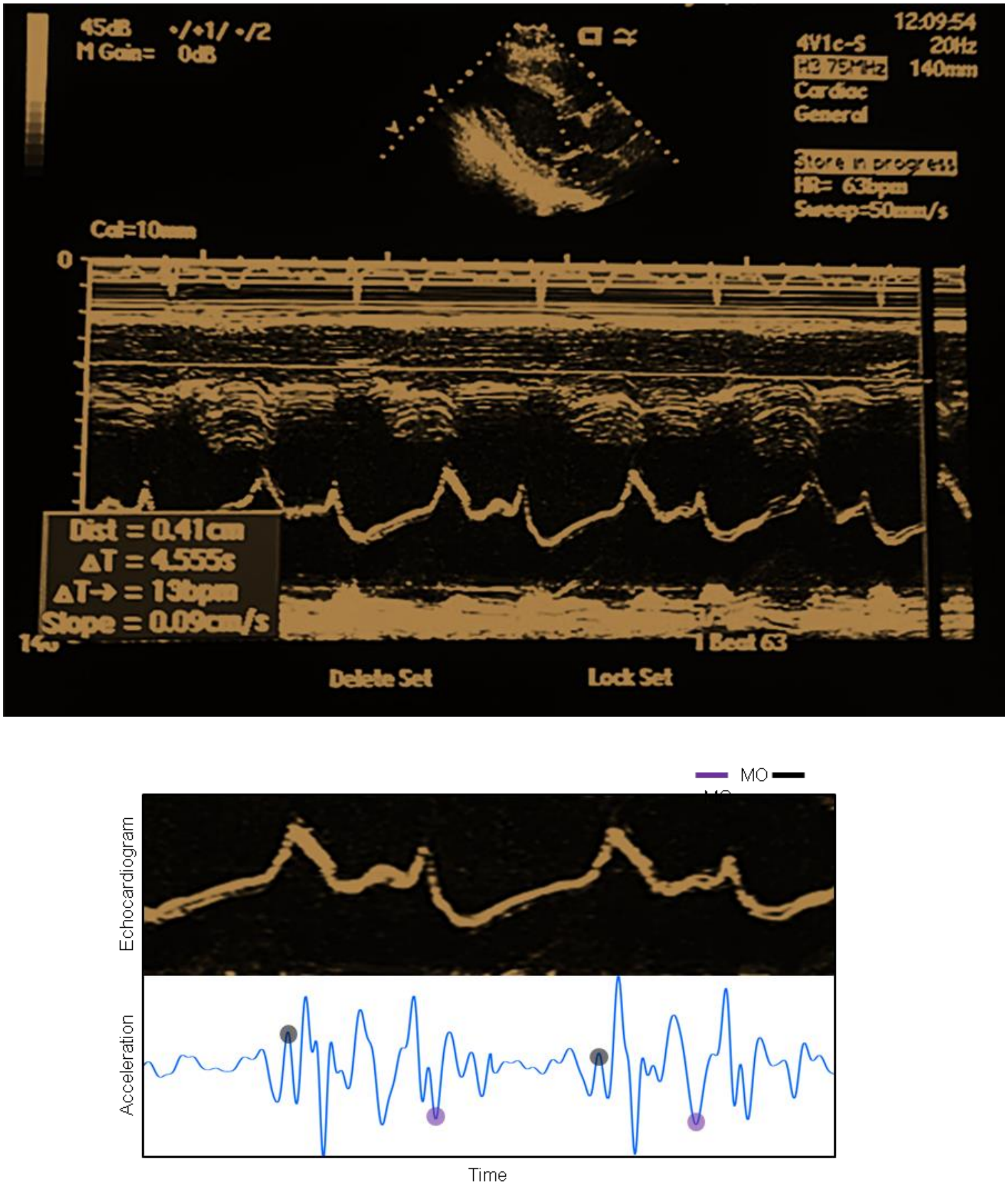

Figure S13. Echocardiogram image of the mitral valve compared to SCG. The detection of the mitral valve opening and closure is possible from the recorded SCG signals.

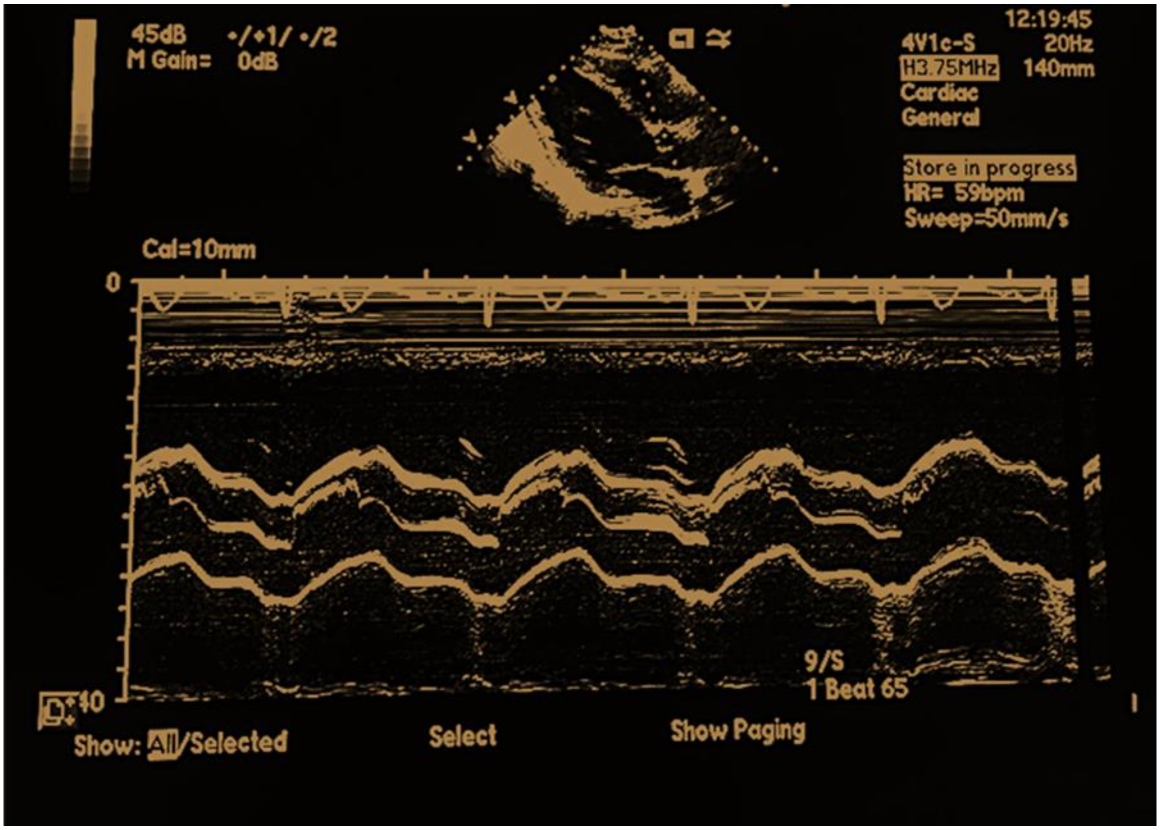

Figure S14. Echocardiogram image of the aortic valve. Echocardiogram data used to validate detection of the aortic valve opening and closing from the SCG signal.

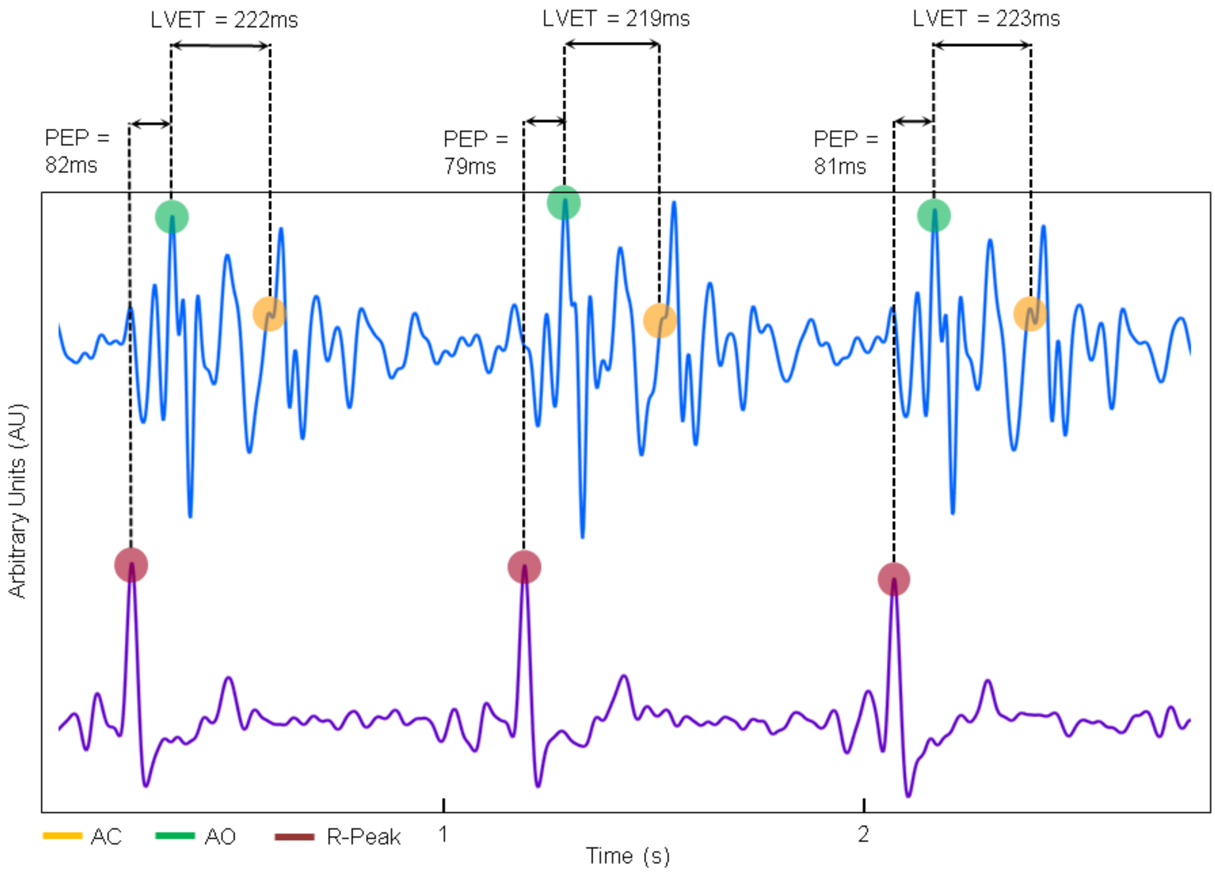

Figure S15. Estimation of PEP and LVET from SCG and ECG. Representative SCG and ECG data with annotated AO, AC and R-peak fiducials. Corresponding PEP and LVET values are reported.

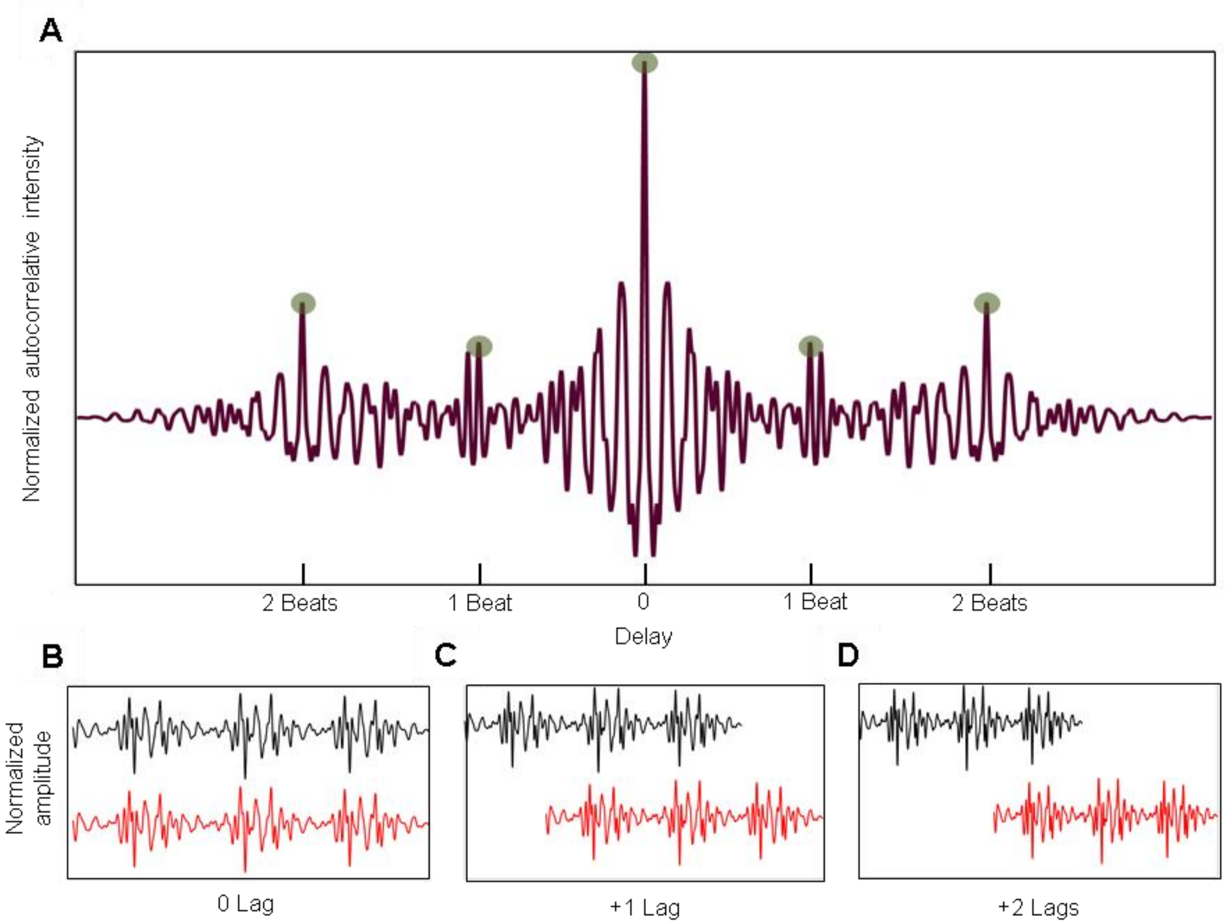

Figure S16. Autocorrelation for the detection of SCG periodicity. (A) SCG autocorrelation identified at the zero, 1 and 2 beat lags. The ratio of the successive lag peaks to the fundamental peak is the autocorellative periodicity. (B-D) Time series SCG signals plotted for zero (B), 1 (C), and 2(D) beat lags.

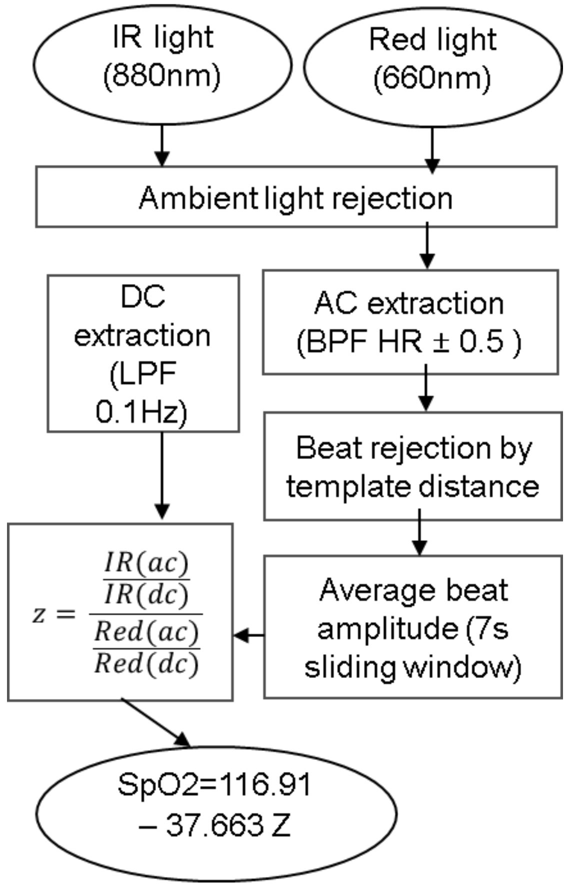

Figure S17. SpO2 detection algorithm.

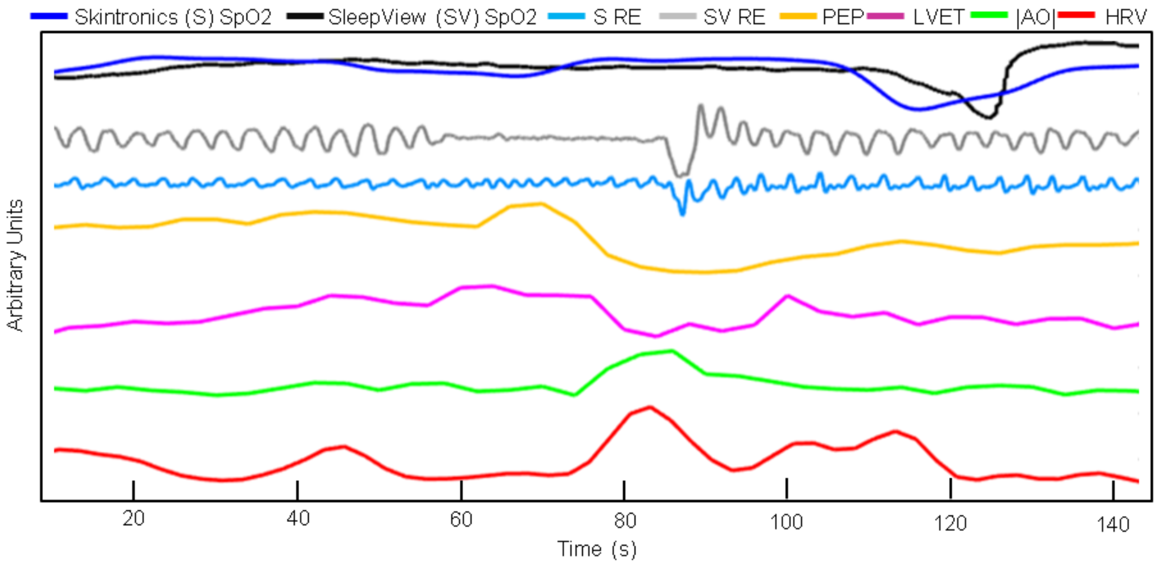

Figure S18. Representative hemodynamic response to a simulated central apnea. Notice that the PEP and LVET clearly decrease, while AO and HRV increase. For this event, venous return exerts a more powerful action than left ventricular afterload.

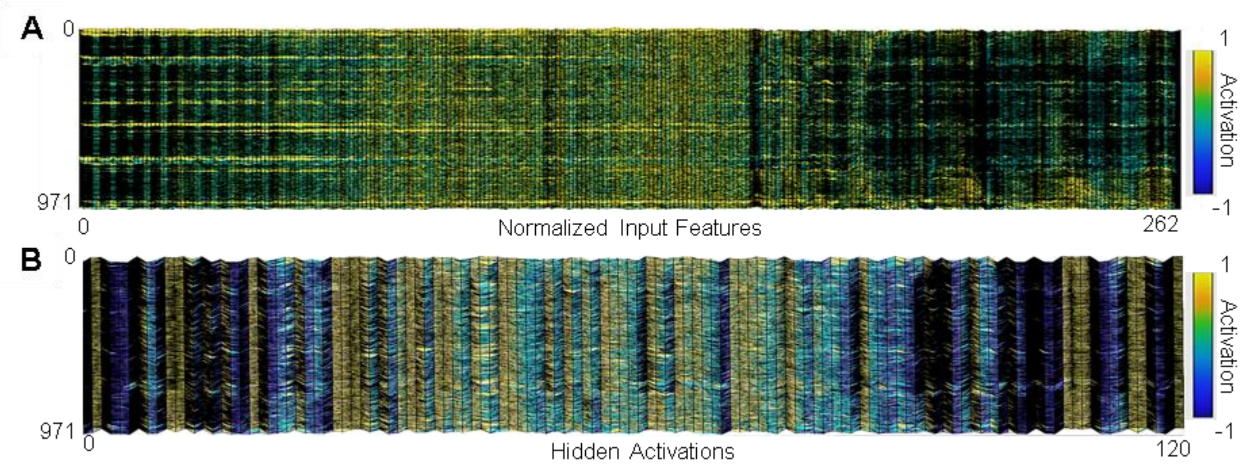

Figure S19. Visualization of the FFNN input features and hidden activations. Surface plot of (A) normalized input features and (B) hidden activations.

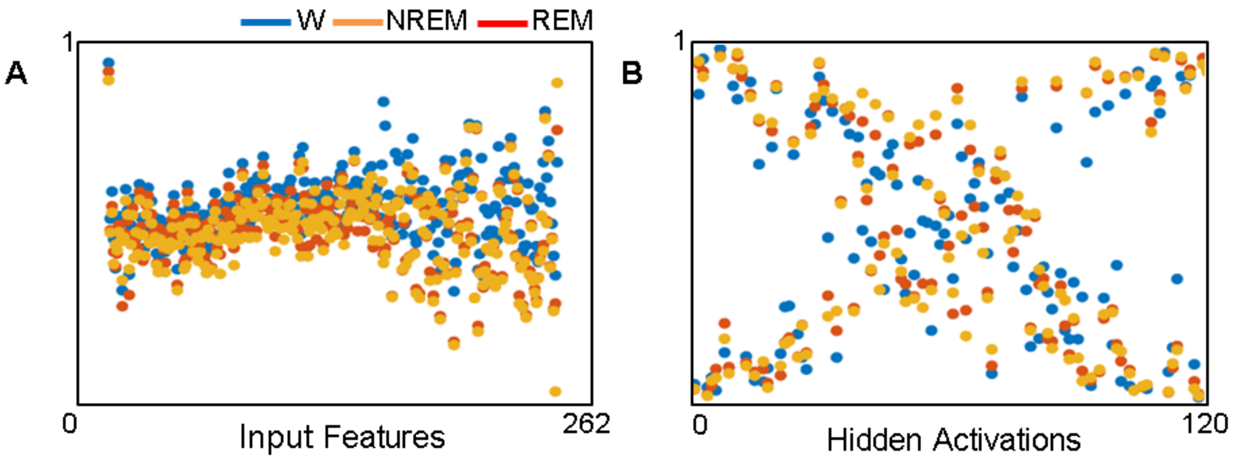

Figure S20. Scatter plot of the FFNN activations. Average magnitudes for each (A) input feature and (B) hidden activation by comparison class.

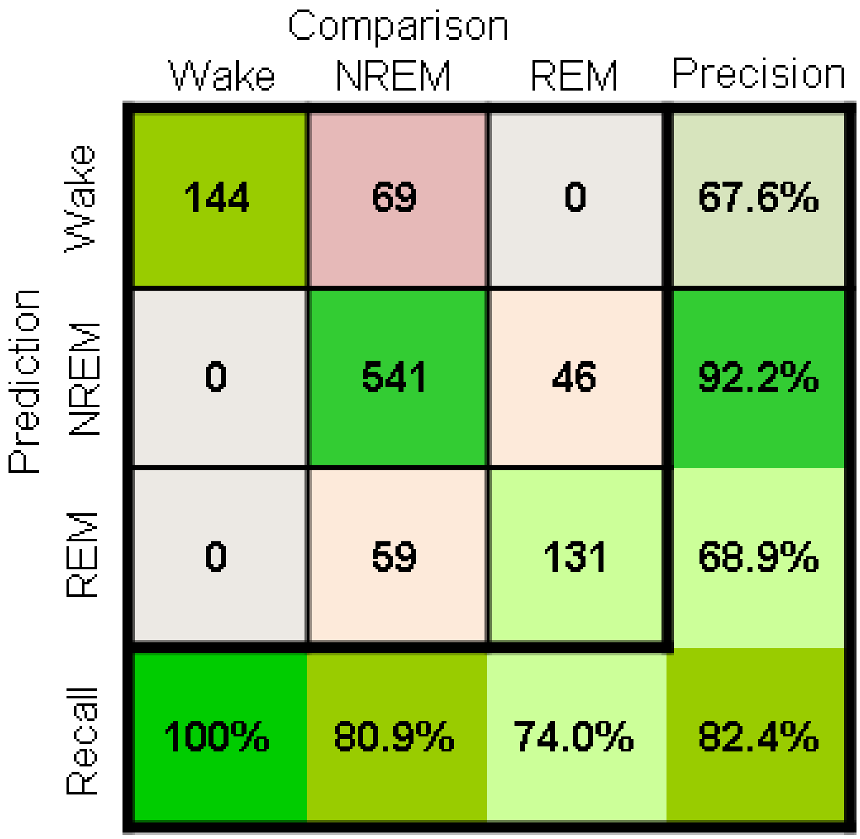

Figure S21. Confusion matrix for classified sleep stages compared to EEG, EOG and EMG collected via the BioRadio.

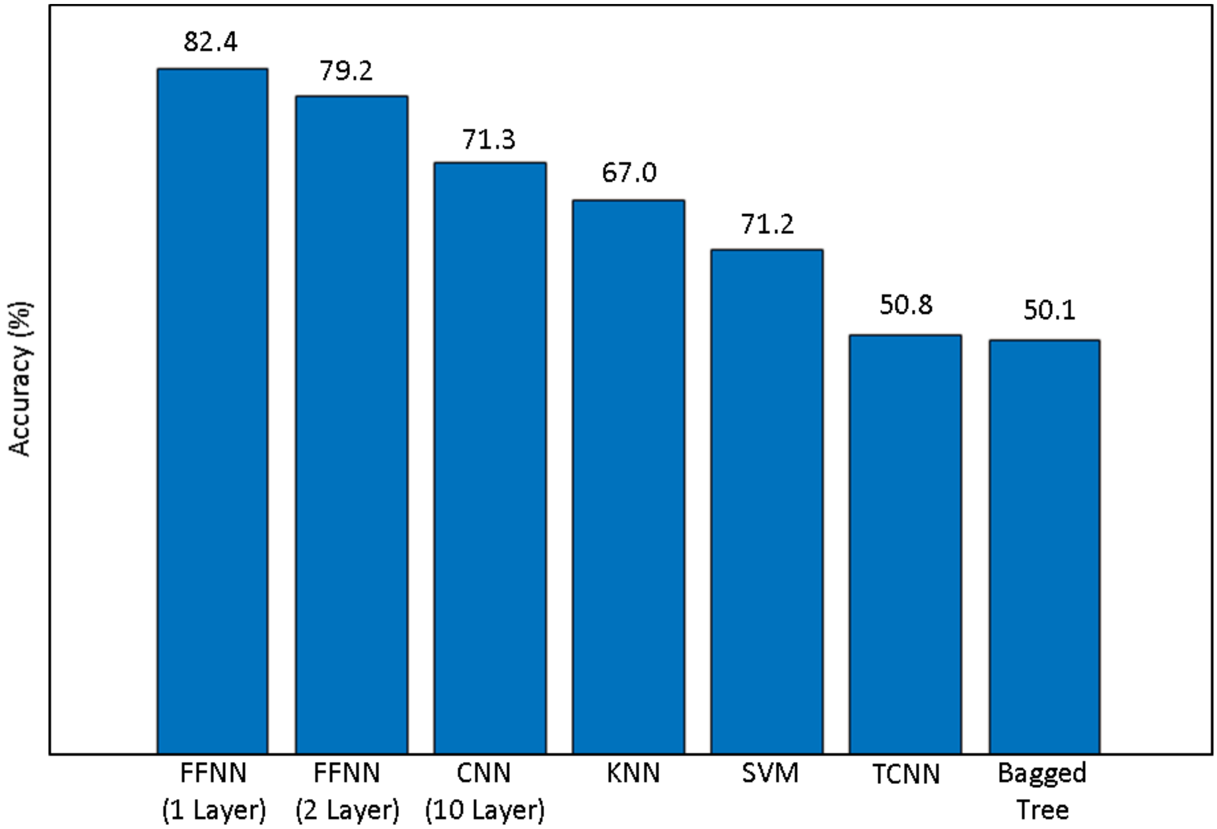

Figure S22. Sleep stage classification accuracy for alternative classifiers. The accuracy derived from the single hidden layer FFNN is compared to additional classifiers, demonstrating that the FFNN yielded the highest accuracy.

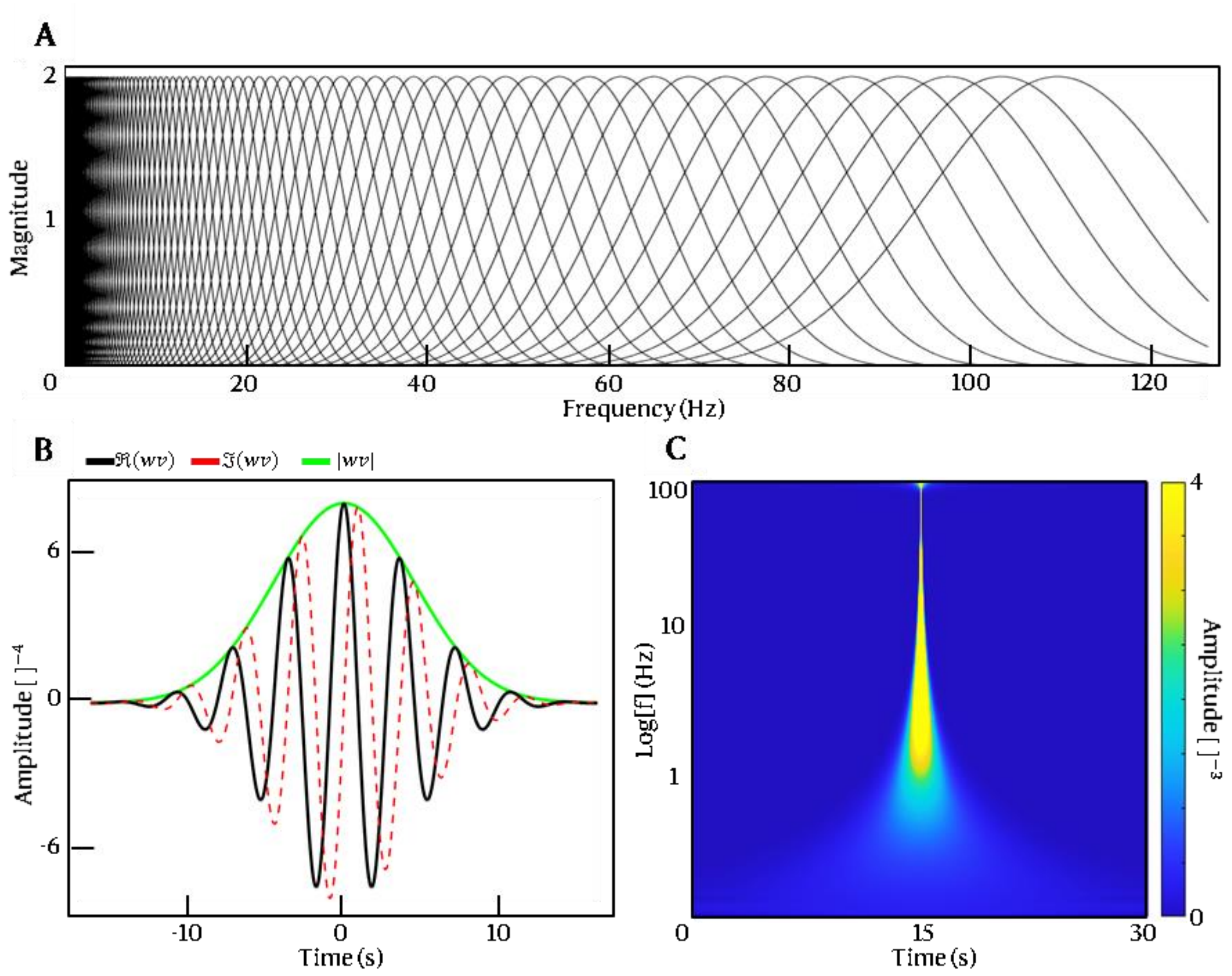

Figure S23. Morse wavelet implementation. (A) Frequency responses for the Morse wavelet filter bank. (B) Time series plot of the complex wavelet under the wavelet magnitude envelope. (C) Scalogram of the impulse response at time 15s demonstrating the maintenance of high temporal resolution until around 1 Hz.

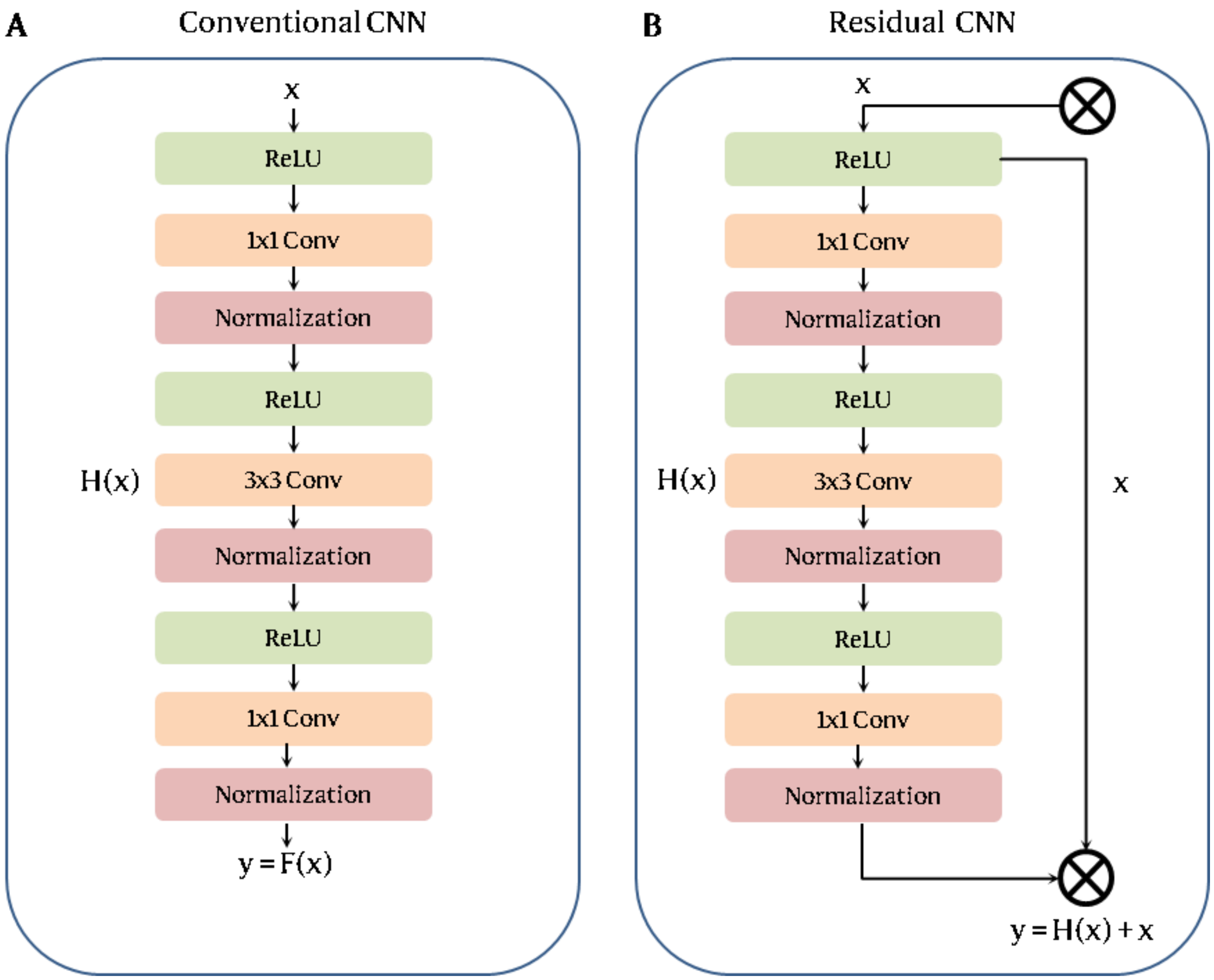

Figure S24. Residual CNN architecture. (A) Conventional CNN architecture where $y_i = H_i(x_i)$ for each layer. (B) Modified residual CNN where $y_i = H_i(x_i) + x_i$.

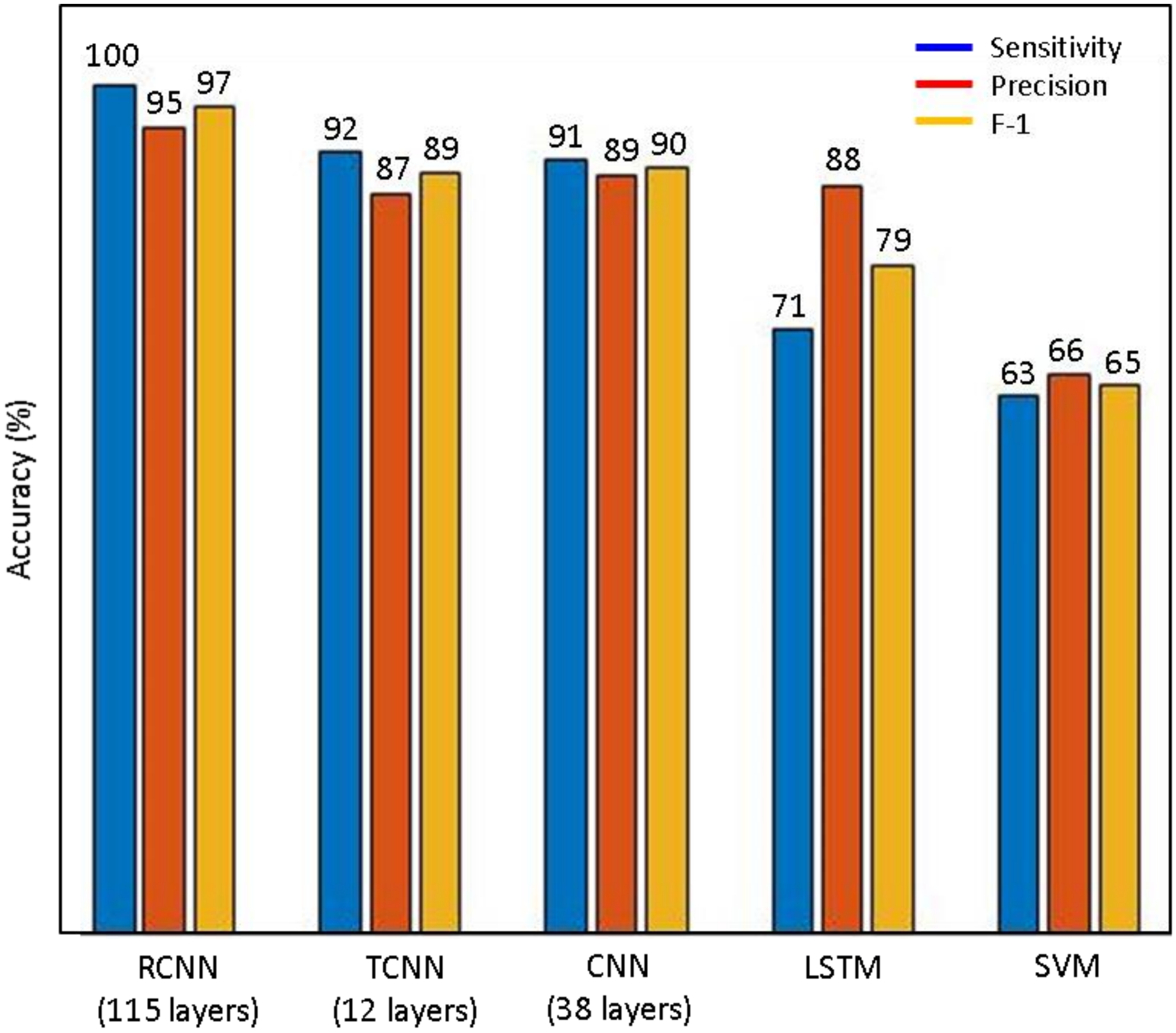

Figure S25. Comparison to alternative apnea classification methods. Sensitivity, precision, and F-1 results for additional classifiers trained on subject data. Here, the RCNN approach yielded the highest accuracy.

Table S1. Sleep staging comparison to published wearable devices.

Subject	Age	BMI	Gender	Race	Dataset Usage	Nights	Stop-Bang/PSQI/ ESS/ISI	OSA
1	31	23.2	M	White	Symptomatic training data	2	Low/9/0/9	Mild (5<x<15)
2	35	29.3	M	White	Symptomatic Validation data	1	Low/7/9/7	Moderate (15<x<30)
3	59	35.2	M	White	Symptomatic training data	2	High/7/4/3	Moderate-Severe (25<x<40)
4	35	27.6	M	Asian	Symptomatic training data/ sleep staging	1	Low/6/3/7	Mild (5<x<15)
5	51	39.4	M	White	Symptomatic training data/ sleep staging	2	Medium/6/4/7	Moderate (15<x<30)
6	50	29.5	F	White	Healthy control/ sleep staging	2	-	-
7	23	22.4	M	White	Healthy control/ sleep staging	3	-	-
8	35	23.8	M	Asian	Healthy control/ sleep staging	1	-	-
9	22	25.8	M	White	Healthy control/ sleep staging	1	-	-
Apnea classification subjects (μ±σ)	42±12	31±6	-	-	-		-	16±12
Sleep staging subjects (μ±σ)	36+11	26±3	-	-	-		-	-

Table S2. Sleep staging F-1 scores by algorithm

	FNNN (1 Layer)	FFNN (2Layer)	CNN	KNN	SVM	TCNN	Bagged Tree
F-1 Wake	80.7%	78.1%	64.4%	56.8%	52.0%	45%	46.1%
F-1 NREM	86.2%	83.4%	76.7%	73.9%	69.5%	62.8%	62.5%
F-1 REM	71.4%	66.0%	61.6%	56.6%	45.4%	7.7%	0%
Micro F-1	82.4%	79.2%	71.3%	67.0%	61.2%	50.8%	50.1%
Weighted F-1	85.4%	82.7%	74.9%	71.4%	67%	60.1%	60.1%

Video S1. Compression of the soft device against a biomimetic skin model.

Video S2. Compression of a rigid comparison device against a biomimetic skin model.

Video S3. Demonstration of real-time signal recording with the soft sternal patch.
